# Mechanical and Electrical Responses and Damage Evolution of Compacted Machine-Made Snow

**DOI:** 10.3390/ma19214511

**Published:** 2026-10-27

**Authors:** Xuan Chen, Enzhao Xiao, Jiashi Li, Jinli Wang, Wenkui Feng, Xiaoqiang Dong

**Affiliations:** 1 College of Civil Engineering, Taiyuan University of Technology, Taiyuan 030024, China; 2 R & D Division of Polar Snow and Ice Runways, Polar Research Institute of China, Shanghai 200129, China; 3 Engineering Management Department, Shaanxi Hydrogen Energy New Energy Co., Ltd., Yulin 719003, China; 4 School of Applied Science, Taiyuan University of Science and Technology, Taiyuan 030024, China

**Keywords:** compacted machine-made snow, low-temperature uniaxial compression, snow mechanics, process apparent resistivity, mechanical properties

## Abstract

**Highlights:**

- Conduct factorial tests to assess temperature, density, and loading-rate effects.
- Identify how temperature and loading rate modify densification strengthening.
- Reveal opposite initial apparent resistivity shifts with cooling and densification.
- Identify strain offsets between peak stress and minimum apparent resistivity.
- Develop a peak-referenced Weibull model to describe local post-peak softening.

**Abstract:**

The relationship between electrical change and load-bearing deterioration remains unclear for dense compacted snow. This study combines full-factorial uniaxial compression tests with concurrent electrical measurements to examine the effects of temperature, density, and loading rate on compacted machine-made snow. Densification increased peak strength, but the magnitude of this gain depended on temperature and loading rate. At −30 °C and 15 mm/min, increasing density from 0.50 to 0.70 g/cm^3^ raised peak strength from 1.072 to 2.544 MPa and reduced initial process apparent resistivity by 62.6%. Cooling also strengthened the snow but increased initial apparent resistivity. Faster loading reduced peak strain, whereas strength varied nonmonotonically. These responses can be interpreted through competition between contact strengthening, stress redistribution, and bond failure. The strain separation between stress peaks and resistivity minima is consistent with contact adjustment continuing during mechanical softening. A peak-referenced Weibull relation described local post-peak softening with a root-mean-square error of 0.041 MPa and R^2^ of 0.920. Adding a nonnegative resistivity rebound term did not improve the local fit. The results distinguish electrical state evolution from mechanical capacity loss and provide a basis for joint assessment of strength, deformation, and resistivity in compacted snow pavements.

## 1. Introduction

Compacted snow forms the bearing layer of temporary polar roads and snow runways, making its response to loading central to their construction and maintenance [1,2,3,4,5]. Processing and compaction improve trafficability by changing the density and structure of the snow layer [6,7,8]. Its performance also depends on the deformation required to mobilize peak strength and the capacity retained afterward. Relating these mechanical changes to an observable response during loading would help connect laboratory strength measurements with the assessment of compacted snow in service.

Density describes the amount of ice per unit volume, but load-bearing capacity also depends on how that ice is connected [9,10,11]. Uniaxial tests on compacted and machine-made snow establish a strong dependence of strength on density and preparation conditions [12,13,14]. Imaging and microstructure-based simulations relate load transfer to grain contacts and their connecting sections [15,16,17,18,19,20]. Recent compression tests on depth hoar identify density as the main strength predictor, with horizontal microstructural dimensions explaining part of the remaining variation [21]. In compacted Antarctic snow, pressure sintering produces a substantial strength gain and improved directional connectivity despite a modest density increase [22]. These results concern different snow structures, yet both distinguish solid content from load-transmitting geometry. This distinction motivates examining dense compacted snow as an evolving contact structure.

Temperature and loading rate determine how that structure responds to force [23,24,25,26]. Time-dependent ice deformation can redistribute local stress, whereas faster loading gives contacts less time to adjust [27]. Recent simulations show that pore space accommodates crystal-scale deformation during snow creep [28]. Models of densely packed snow relate temperature and rate effects to the deformation and failure of ice bridges [29], while slow-compression experiments reveal a grain-size dependence of the active deformation mechanism [30]. Higher-rate tests address a different balance between adjustment and rupture [31]. Hodgdon et al. prepared snow in cylinders 50 mm in diameter and 155 mm high and investigated unconfined compression above 0.01 s^−1^ [32]. They identified scaled bond contact area as a strong predictor of strength.

The compression curve reflects the balance between contact development and loss of load-bearing capacity [33,34]. Grain rearrangement and pore contraction can strengthen some regions while highly stressed bonds fail in others. Discrete-element studies represent this coexistence of contact creation and rupture [16,35]. Particle image velocimetry also resolves nonuniform deformation during compaction of unsintered snow layers [36]. These observations explain why macroscopic softening need not mark the end of local compaction. A complementary signal acquired during loading can reveal whether another aspect of the material response continues to evolve after load capacity begins to fall.

Electrical measurements provide that complementary observation, with sensitivity to both structure and charge transport [37]. Snow conductivity depends on temperature and impurities, while the alternating-current response also depends on frequency and polarization [38,39,40,41]. Compression changes contact areas and gaps between grains, which can alter the measured electrical path at fixed temperature. Initial apparent resistivity and its subsequent change therefore have different meanings. The former characterizes the electrical condition before loading; the latter follows the response as compression proceeds. Comparing stress and process apparent resistivity at the same strain can reveal how their characteristic changes are related. In particular, locating the resistivity minimum relative to the stress peak helps distinguish electrical adjustment during net hardening from adjustment that continues into softening.

A remaining gap is the connection between these electrical changes and mechanical deterioration in dense compacted snow. Structural studies explain how density and ice deformation influence strength, but do not establish which electrical features correspond to loss of load-bearing capacity. Temperature and compaction can also affect the two signals through different processes. Their correspondence therefore needs to be examined under controlled conditions. A further question is whether an electrical descriptor explains stress loss beyond the shared dependence on strain. Continuum damage mechanics represents deterioration through a decreasing effective load-bearing fraction [42], and a Weibull distribution describes dispersed failure thresholds [43]. Snow fiber-bundle models represent load-bearing units that fail and redistribute load [44,45]. Recent compressive formulations also incorporate strengthening and rebonding [46,47,48]. These developments provide a mechanical basis for testing the additional contribution of resistivity rebound to a local softening relation.

This study examines these questions through concurrent mechanical and electrical measurements on compacted machine-made snow. A factorial program covers temperatures from −50 to −10 °C, densities from 0.50 to 0.70 g/cm^3^, and loading rates from 5 to 30 mm/min. Comparisons at fixed conditions relate peak strength and deformation to the initial electrical level and its evolution during loading. The strain positions of the stress peak and resistivity minimum are then compared. A peak-referenced Weibull relation describes local post-peak softening, and nonlinear regression evaluates an additional term derived from resistivity rebound history.

## 2. Materials and Methods

### 2.1. Snow Preparation and Specimen Fabrication

Machine-made snow was produced with an NP-4000 snowmaker (Manufactured by Henan Jin’an Machinery Technology Co., Ltd., Zhengzhou, China) at approximately −5 °C and stored in a temperature-controlled chamber at −18 °C. The initial snow density was approximately 0.30–0.32 g/cm^3^. The recorded water-content values ranged from approximately 2.86% to 2.91% across test batches. Deionized water was used to minimize the influence of ions in the preparation water on subsequent tests. Before specimen fabrication, the loose source snow was passed through a 2 mm sieve in the cold laboratory. The snow required for each specimen was divided into five equal portions by mass and placed in the cylindrical mold in successive layers. Each layer was statically compacted before the next was added. The specimens were 50 mm in diameter and 100 mm in height. After demolding, they were conditioned at the target temperature for 30 min.

### 2.2. Experimental Design

This study varied temperature, nominal density, and loading rate in a full-factorial matrix. It included nine temperatures from −50 to −10 °C at 5 °C intervals, five nominal densities from 0.50 to 0.70 g/cm^3^ at 0.05 g/cm^3^ intervals, and six loading rates from 5 to 30 mm/min at 5 mm/min intervals. Four separately prepared specimens were tested at each condition. Table 1 summarizes the controlled factors and measurement settings. The full-factorial layout supports comparisons at fixed levels of the other factors and provides a basis for testing interactions.

### 2.3. Synchronized Resistivity Measurement

**Figure 1:**
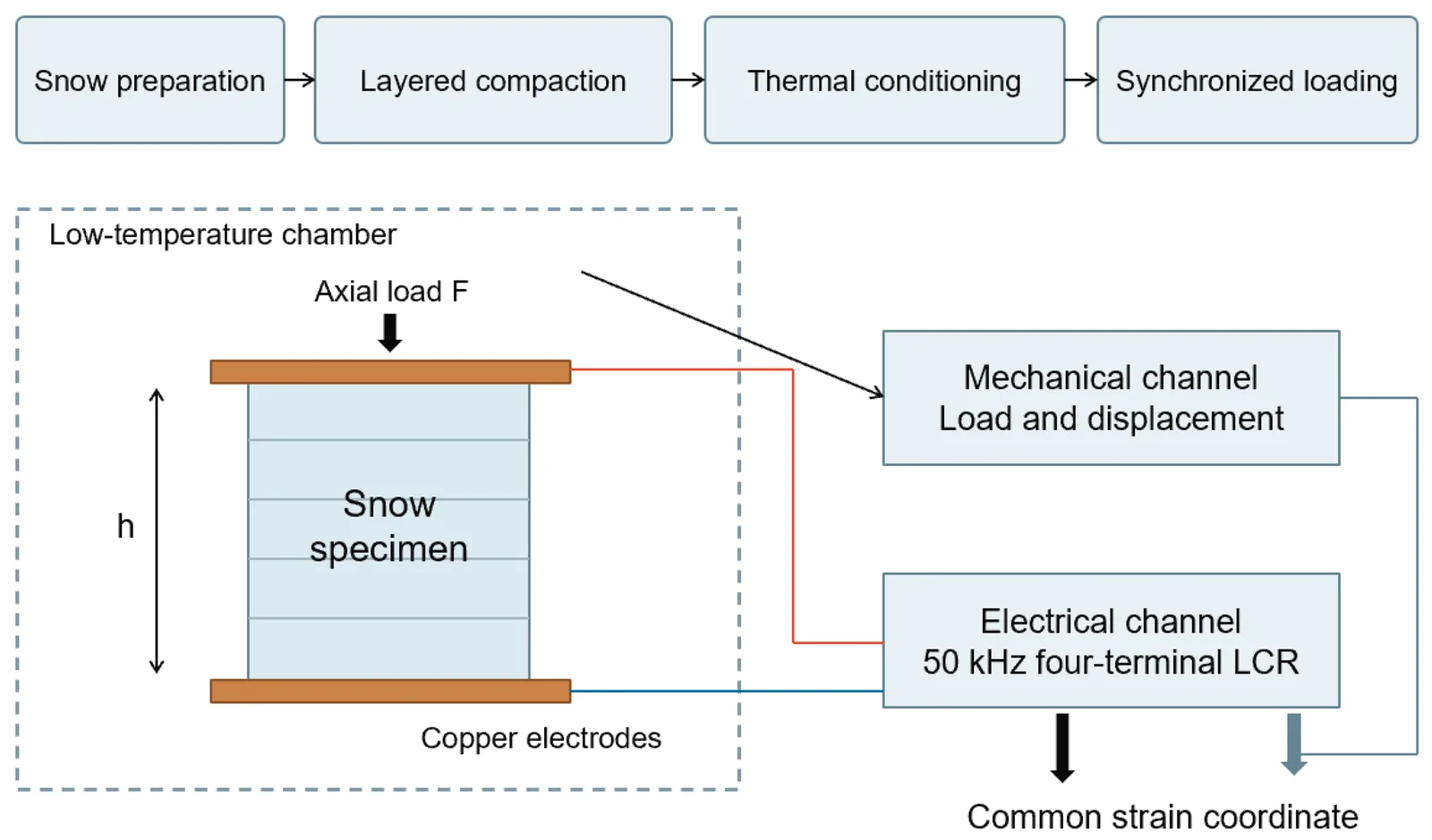
Preparation of machine-made snow and the arrangement for concurrent low-temperature mechanical and electrical measurements. The diagram shows the two copper plates and their instrument connections.

Figure 1 summarizes snow preparation and the low-temperature mechanical and electrical test arrangement. Uniaxial compression used a WDW-T300 testing machine (Manufactured by Jinan Tianchen Testing Machine Manufacturing Co., Ltd., Jinan, China) with a 30 kN load cell and an indicated-value error of ±0.5%. A TH2828A LCR meter (Changzhou Tonghui Electronics Co., Ltd., Changzhou, China) measured the electrical response at 50 kHz. Copper-plate electrodes contacted the upper and lower specimen faces. Each plate carried a current lead and a voltage-sensing lead. This four-terminal connection reduced lead and terminal contributions while retaining both electrode–snow interfaces in the measured path. A thin layer of low-temperature conductive paste was applied at the interfaces to increase the area of electrical contact. Load, displacement, and electrical signals were recorded during the same loading process.

Axial load, compressive displacement, and equivalent resistance describe the concurrent mechanical and electrical responses. Engineering strain is calculated as ε = Δh/h_0_ from axial shortening. Engineering stress and process apparent resistivity are calculated using Equations (1) and (2), respectively:

(1) $$\sigma =\frac{F}{A_{0}}\;$$

(2) $$\rho =\frac{RA_{0}}{L}$$

Here, *σ* is engineering stress and *F* is axial load. *A*_0_ is the initial specimen cross-sectional area used in both equations. In the strain definition, Δh is axial shortening and h_0_ is the initial height of 100 mm. *R* is the equivalent resistance component reported by the LCR meter at 50 kHz. *L* denotes the electrode separation used in the electrical geometric conversion. The measured quantity includes the snow specimen and both plate–snow interfaces. It is therefore termed process apparent resistivity and is expressed in Ω·m.

**Figure 2:**
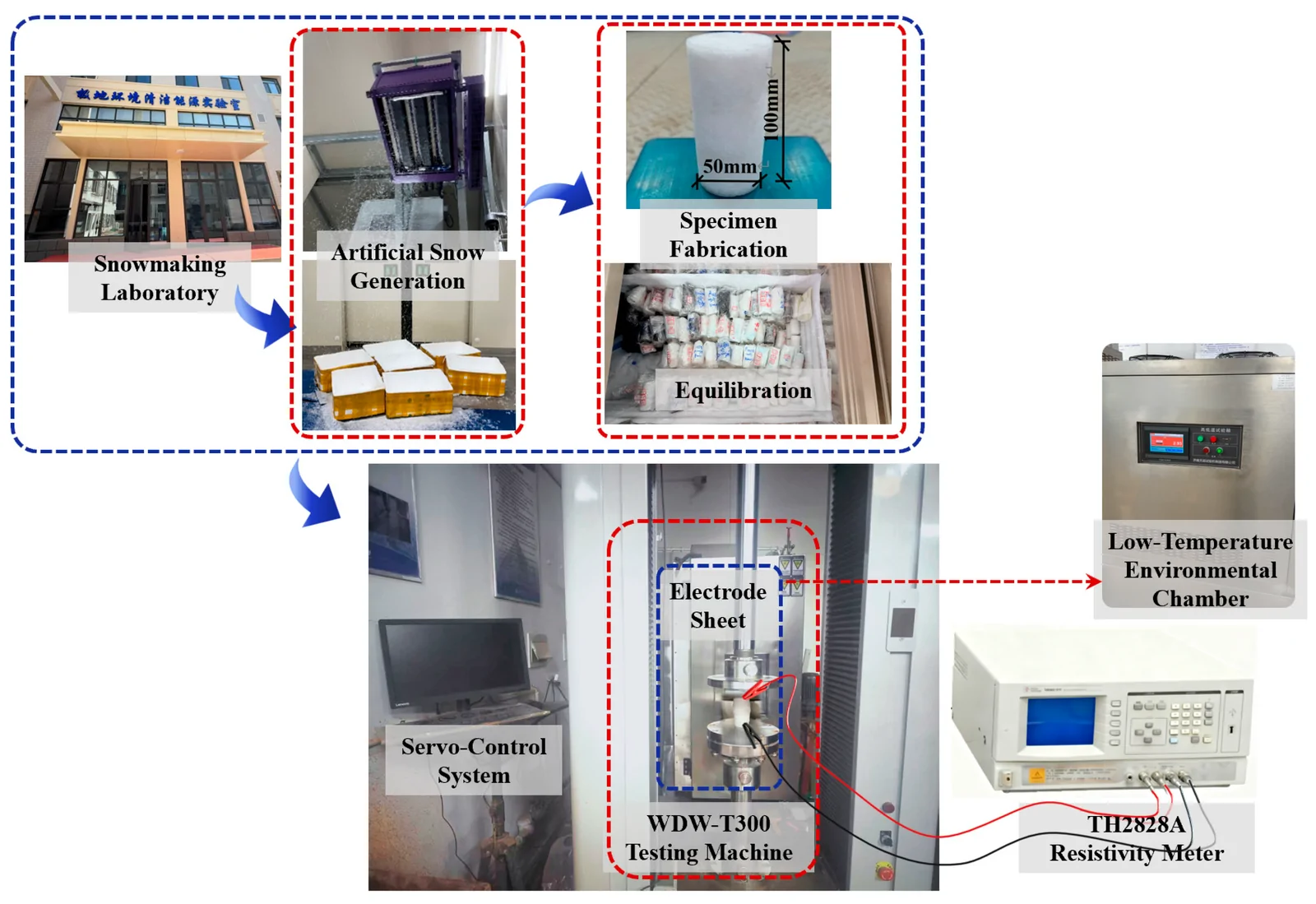
Experimental workflow and laboratory setup.

A frequency of 50 Hz was selected for testing and recording because it produced the most stable resistivity readings during the experiments. Mechanical and electrical records were aligned on engineering strain. Each electrical observation retained its strain coordinate, and stress was linearly interpolated at those coordinates for the local post-peak analysis. The displayed responses summarize four specimens per condition. Averaging specimen-specific features and extracting features from an averaged trajectory are distinct operations. The archive contains strain-indexed electrical values rather than time stamps. It therefore supports comparison in strain, without a measured temporal lag or separate temporal sampling frequency. The local calibration uses N_ε_ = 7 positions on one condition-level mean post-peak response. These positions are distinct from the n_rep_ = 4 specimens summarized by that response. Only the final two positions have positive rebound history. The experimental workflow is shown in Figure 2.

## 3. Results and Discussion

### 3.1. Load-Bearing Stages and Factor-Dependent Strength

#### 3.1.1. Compaction-Induced Hardening and Post-Peak Softening

**Figure 3:**
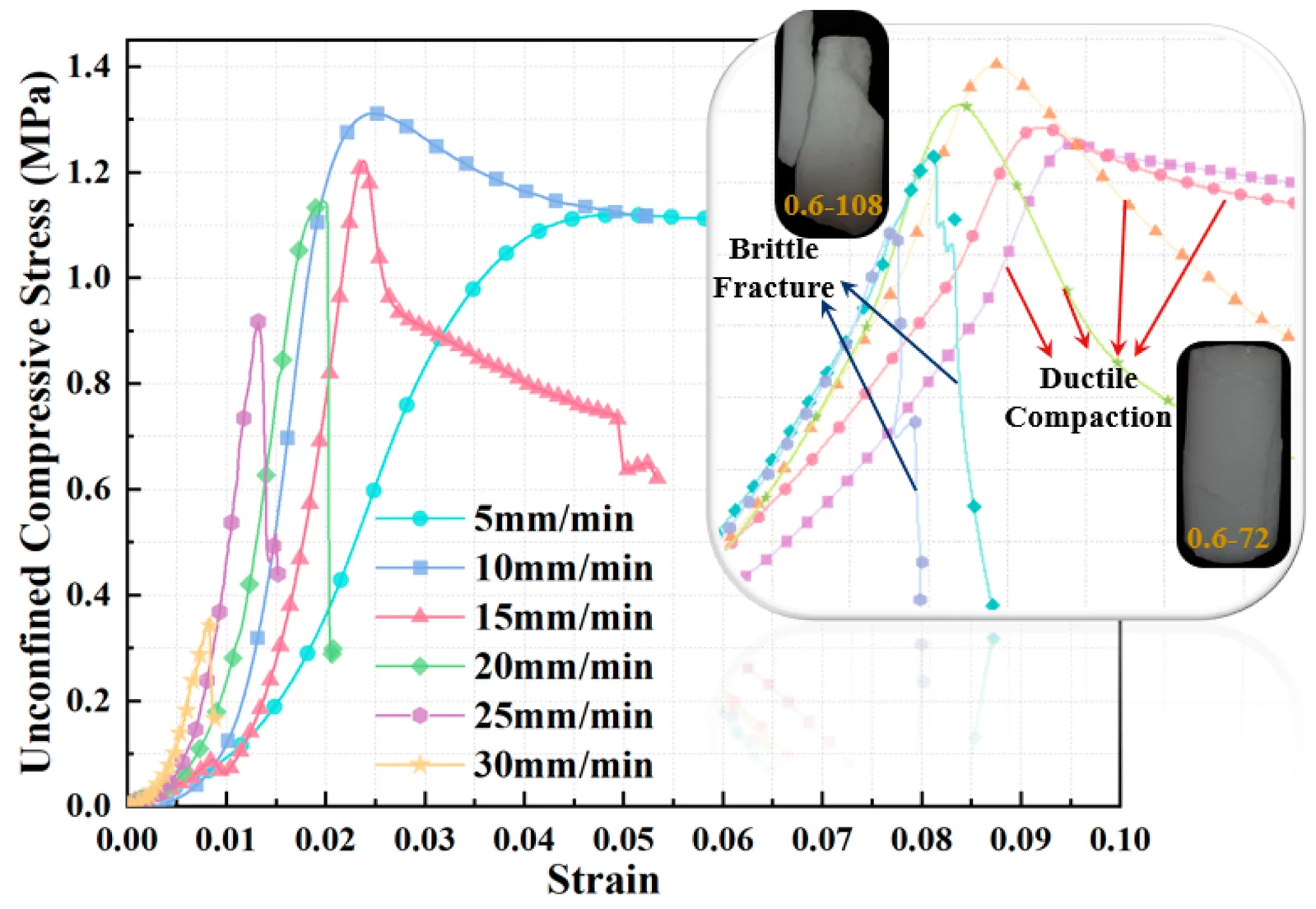
Uniaxial compressive responses of compacted machine–made snow at different loading rates.

Figure 3 shows that loading rate changes not only the load-bearing level of compacted snow but also its pre-peak deformation and post-peak loss of capacity. At 5 mm/min, the curve reaches a plateau of approximately 1.1 MPa after a relatively long compression interval. At 10 mm/min, stress reaches approximately 1.3 MPa at a strain of about 0.025 and then decreases gradually. In contrast, the curves at 20 and 25 mm/min exhibit sharp peaks at smaller strains, followed by pronounced stress drops. At 15 mm/min, the initial rapid post-peak decline is followed by gradual softening. These differences show that higher peak strength does not necessarily coincide with more stable post-peak load support. The compressive behavior of compacted snow therefore needs to be interpreted through both contact strengthening and the failure of load-bearing contacts.

The progressive increase in pre-peak slope can be interpreted in terms of pore compression and the development of effective load-transmitting contacts. During initial loading, part of the axial displacement accommodates grain rearrangement and seating at the specimen ends, so macroscopic stress increases slowly. Continued compression reduces the gaps between grains, tightens existing contacts, and brings new contacts into load transmission. The stress increase per unit strain consequently becomes larger. Using compression tests and X-ray computed microtomography, Wang and Baker found that sintered snow with greater connectivity and larger bonds exhibited faster stress growth during deformation. Their observations provide direct evidence for hardening associated with an improved contact structure [15].

Sustained load support at a low loading rate is consistent with time-dependent deformation of the ice phase and adjustment of grain contacts. Under slower loading, viscoplastic deformation of stressed ice bridges can relieve local stress concentrations. Grains also have more time to adjust their contacts, allowing load to be progressively redistributed to other parts of the structure. Simulations by Védrine et al. using real three-dimensional microstructures showed that pore space accommodates incompatible deformation between ice crystals and that crystal-scale mechanisms contribute substantially to macroscopic viscoplasticity [28]. Compression therefore need not immediately produce a through-going fracture. The extended deformation interval and subsequent plateau at 5 mm/min can be interpreted as continued effective load transmission during structural deformation. The small stress changes near the plateau reflect a temporary balance between load capacity and deformation, rather than the cessation of structural evolution.

The abrupt stress drops at higher loading rates are more consistent with concentrated failure of critical load-bearing contacts. At 20 and 25 mm/min, rapid stress growth changes to a pronounced decline near the peak, indicating a substantial loss of capacity over a small additional strain interval. A possible mechanism is that local contact failure transfers load to neighboring contacts, increasing their stress and promoting further failure. Macroscopic axial compression does not mean that every ice bridge is subjected to pure compression. Contact orientation and grain motion can impose shear and bending on local bonds and generate tensile stresses.

The nonmonotonic peak-strength response further shows that faster loading can have opposing effects on load capacity. The peak at 10 mm/min exceeds that at 5 mm/min, but several curves show lower peaks and faster post-peak declines as loading rate increases further. Wang et al. likewise observed an initial increase followed by a decrease in strength with loading rate in uniaxial tests on compacted snow, accompanied by a transition from plastic deformation to brittle failure [12].

The capacity retained after the peak provides another distinction between these failure processes. The curves at 10 and 15 mm/min retain load-bearing capacity after reaching their maxima, showing that the loss of peak capacity does not eliminate all load transmission. Surviving bonds, newly established contacts, and intergranular friction can continue to resist further compression. Discrete-element descriptions that allow new contacts to form as existing contacts fail, together with compressive models incorporating both strengthening and damage, provide a mechanistic basis for this gradual softening [16,46]. For curves with a distinct peak, the peak position can therefore be interpreted as the transition from net growth to net decline in overall load capacity. Peak strength describes the load-bearing level at that transition, peak strain describes the deformation required to reach it, and the post-peak curve describes continued load transmission after structural instability. Together, these quantities provide the mechanical basis for comparing temperature, density, and loading-rate effects below.

#### 3.1.2. Temperature, Density, and Loading-Rate Effects on Peak Response

Temperature, density, and loading rate influence different parts of the compression mechanism. Density changes the amount and arrangement of load-bearing material, whereas temperature affects deformation of the ice contacts. Loading rate determines the time available for stress redistribution. Comparing strength together with peak strain therefore distinguishes an increase in load capacity from a change in the deformation needed to mobilize it.

**Figure 4:**
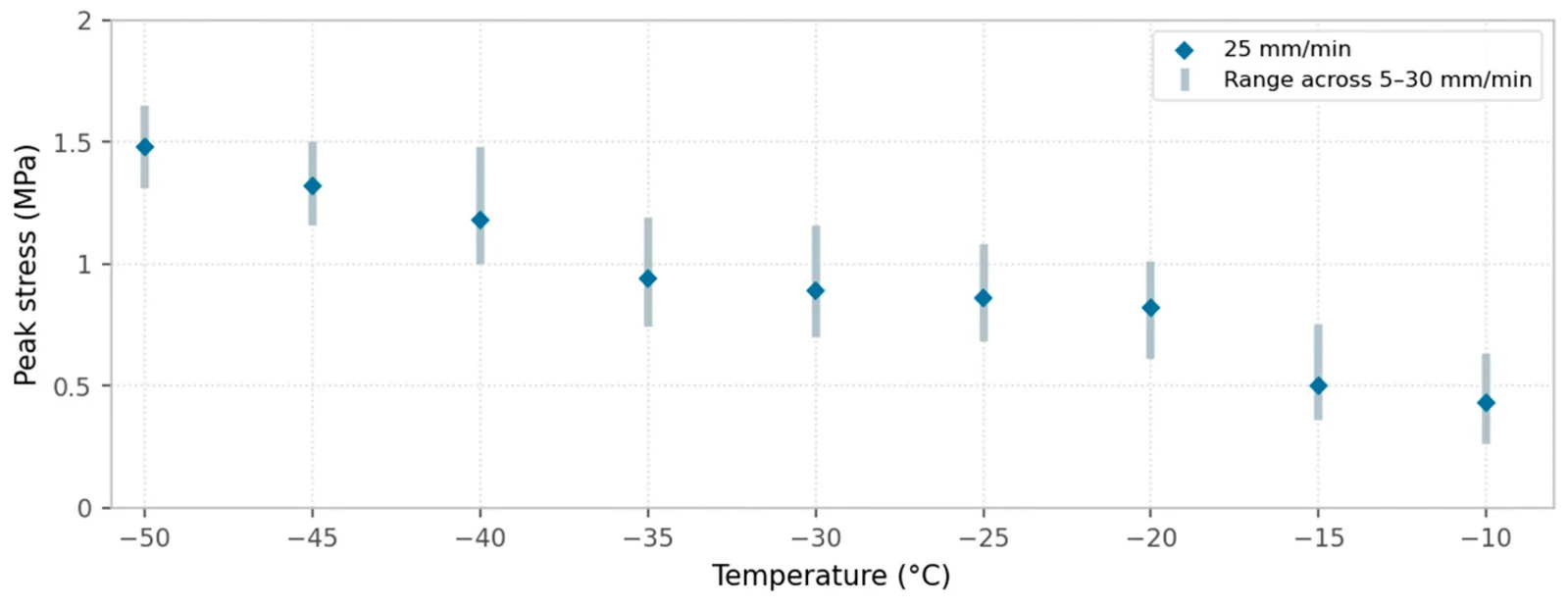
Peak strength across temperature at 0.50 g/cm^3^. Gray ranges span the six loading-rate means from 5 to 30 mm/min; diamonds denote the 25 mm/min result. The ranges describe variation across loading rates. Stress is in MPa, and temperature is in °C.

Figure 4 shows a reduction in peak strength with warming at a density of 0.50 g/cm^3^. At 25 mm/min, strength decreased from 1.438 MPa at −50 °C to 0.419 MPa at −10 °C, a reduction of approximately 70.9%. This interval-based measure expresses thermal sensitivity relative to the starting strength. Mechanically, warming facilitates time-dependent deformation at ice contacts. Slip and creep can redistribute concentrated stress during loading, reducing the stress retained by the structure. Miot et al. reproduced temperature-dependent stress through the viscous response and failure of ice bridges [29]. Védrine et al. also showed that pore space accommodates crystal-scale deformation during snow creep [28]. These results support interpreting the present temperature trend through contact deformation and stress relaxation. The overlap of the cross-rate strength ranges further shows that temperature and loading duration must be considered together, as in the compacted-snow tests of Wang et al. [12].

**Figure 5:**
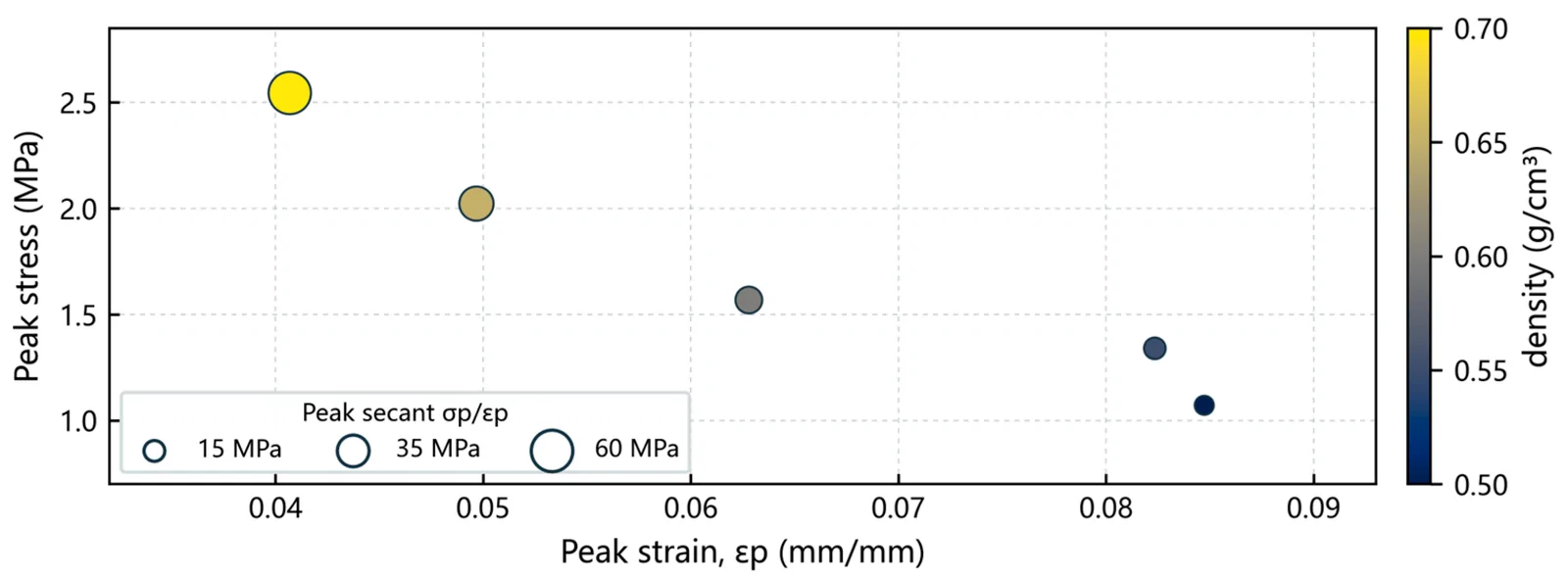
Peak strength and peak strain across density at −30 °C and 15 mm/min. Color denotes density in g/cm^3^, and bubble area represents K_p_ = σ_p_/ε_p_ in MPa. Stress is in MPa, and strain is dimensionless.

Figure 5 links the density-related strength gain to a shorter deformation interval before the peak. At −30 °C and 15 mm/min, increasing density from 0.50 to 0.70 g/cm^3^ raised peak strength from 1.072 to 2.544 MPa and reduced peak strain from 0.084 to 0.041. The strength ratio was 2.373, compared with a density ratio of 1.400. Consequently, strength divided by density increased by approximately 69.5%. The gain thus exceeded the increase in material mass per unit volume. A contact-based explanation is that compaction enlarges effective load-transmitting sections and increases the number of contacts available for load sharing. Reduced pore space also limits further grain rearrangement, explaining the lower peak strain. This interpretation is also consistent with the findings of Xiao et al. [22]. Together, the results link density-related capacity gains to how the ice skeleton carries load.

**Figure 6:**
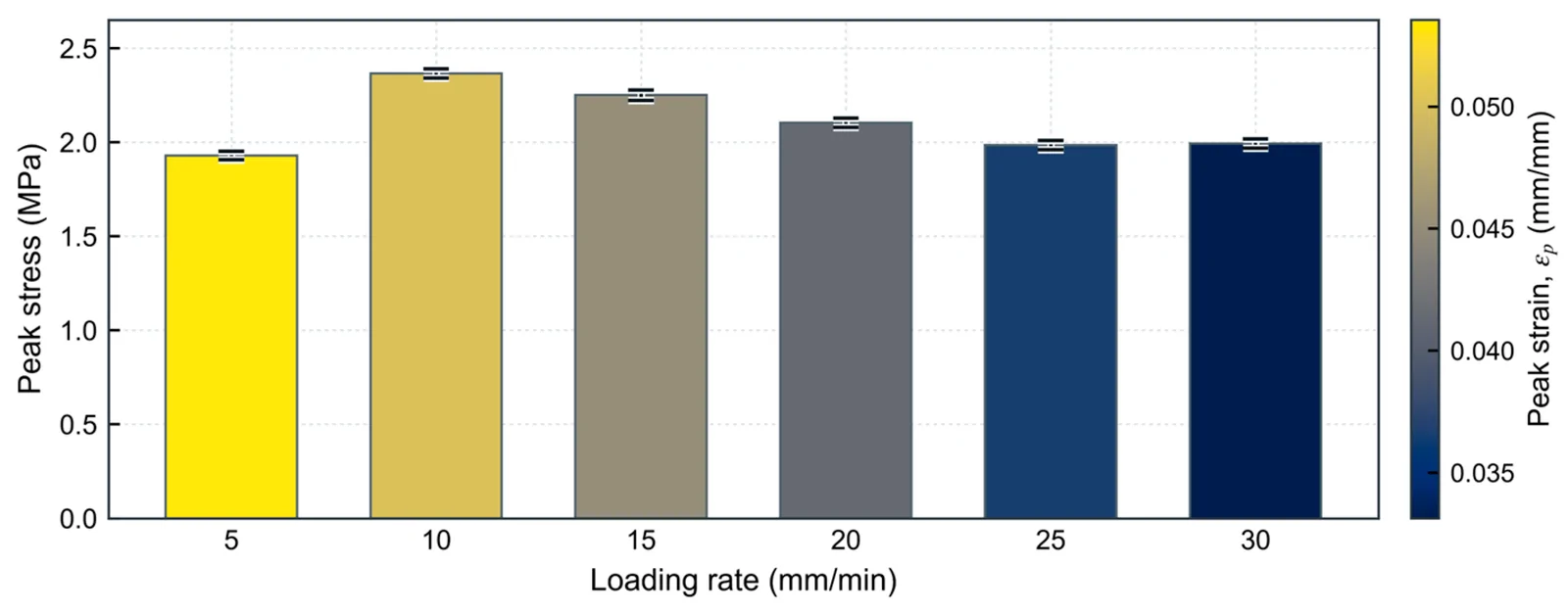
Peak strength across loading rate at 0.65 g/cm^3^ and −40 °C. Bar height gives strength in MPa, and color denotes peak strain. Loading rate is in mm/min.

Figure 6 shows a nonmonotonic strength response to loading rate at 0.65 g/cm^3^ and −40 °C. Peak strength reached 2.339 MPa at 10 mm/min, whereas peak strain decreased from 0.054 to 0.033 between 5 and 30 mm/min. The 38.9% reduction in peak strain shows that faster loading brought forward the loss of net hardening without continuously increasing capacity. At lower rates, contact deformation and stress relaxation reduce the load retained by the structure. Moderate acceleration limits this relaxation and can increase strength. Further acceleration also restricts redistribution around highly stressed contacts, favoring concentrated failure over gradual adjustment. Wang et al. observed a comparable strength maximum in compacted snow, with the associated rate between 10 and 30 mm/min [12]. Differences in temperature and initial contacts can shift this balance. Fiber-bundle models describe the same competition through rupture and rebonding timescales [44], while Xiao et al. explicitly included compression-induced strengthening and load-dependent rebonding [46].

#### 3.1.3. Densification Strengthening Under Three-Factor Interaction

**Figure 7:**
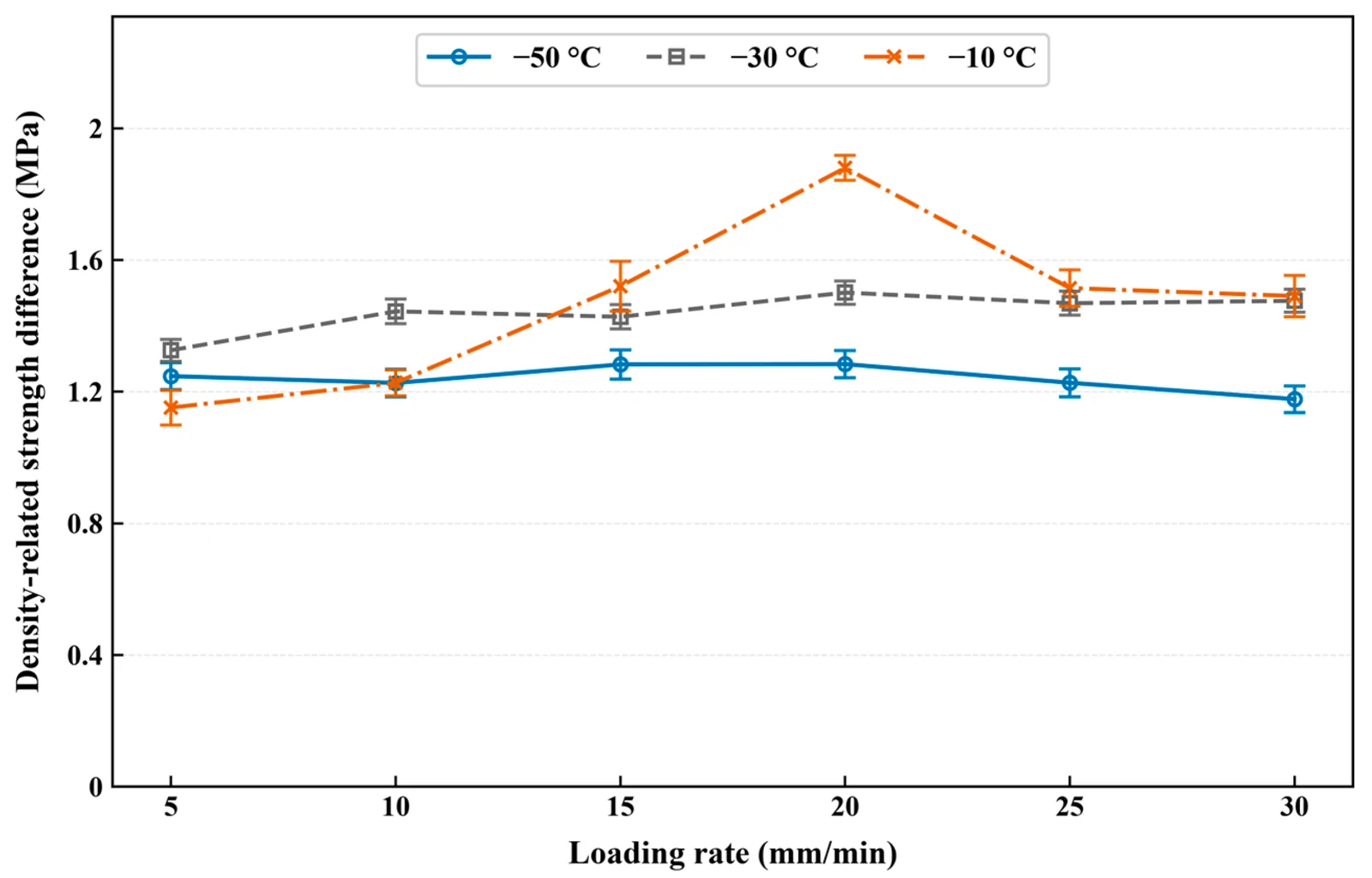
Density-related peak-strength difference across loading rate at different temperatures.

Figure 7 shows that the strength gain from densification is not constant and that its response to loading rate changes with temperature. Relative to −30 °C and 5 mm/min, the three-way interaction contrast at −10 °C reaches approximately 0.55 MPa at 20 mm/min, whereas the corresponding value at −50 °C is approximately −0.14 MPa. Thus, when loading rate increases from 5 to 20 mm/min, the strengthening associated with increasing density from 0.50 to 0.70 g/cm^3^ increases more at the warmer temperature than at the reference temperature, but less at the colder temperature. These opposite contrast directions indicate that temperature modifies the density–rate relationship; they do not mean that warming directly increases strength or that cooling weakens the material.

The mechanical role of density is expressed first through the number and arrangement of load-bearing contacts. At −30 °C and 15 mm/min, densification increased peak strength from 1.072 to 2.544 MPa while reducing peak strain. This combination reflects greater load capacity with less pre-peak deformation due to rearrangement. The result is consistent with the identification of density as an important structural variable by Chen et al. [49] and Xiao et al. [22]. Densification strengthening therefore involves not only an increase in ice content but also larger effective load-transmitting sections and improved local load sharing.

With a further increase in loading rate, the interaction contrast at −10 °C falls from its maximum near 20 mm/min, indicating that the additional densification benefit relative to the reference condition does not continue to increase. Faster loading may limit stress relaxation while also shortening the time available for load redistribution after local contact failure. Concentrated failure of critical connections can then increasingly offset structural strengthening. The compressive fiber-bundle model of Xiao et al. includes both densification strengthening and rebonding and shows that the fraction of viscous deformation varies with loading rate, density, and sintering time [46]. This supports interpreting the nonmonotonic interaction response through competition between strengthening and failure, rather than treating increasing rate as a uniformly strengthening influence. Density is therefore an important control on the load-transmitting structure, while temperature and loading rate jointly determine how much of that structural advantage is realized as additional peak load capacity.

**Figure 8:**
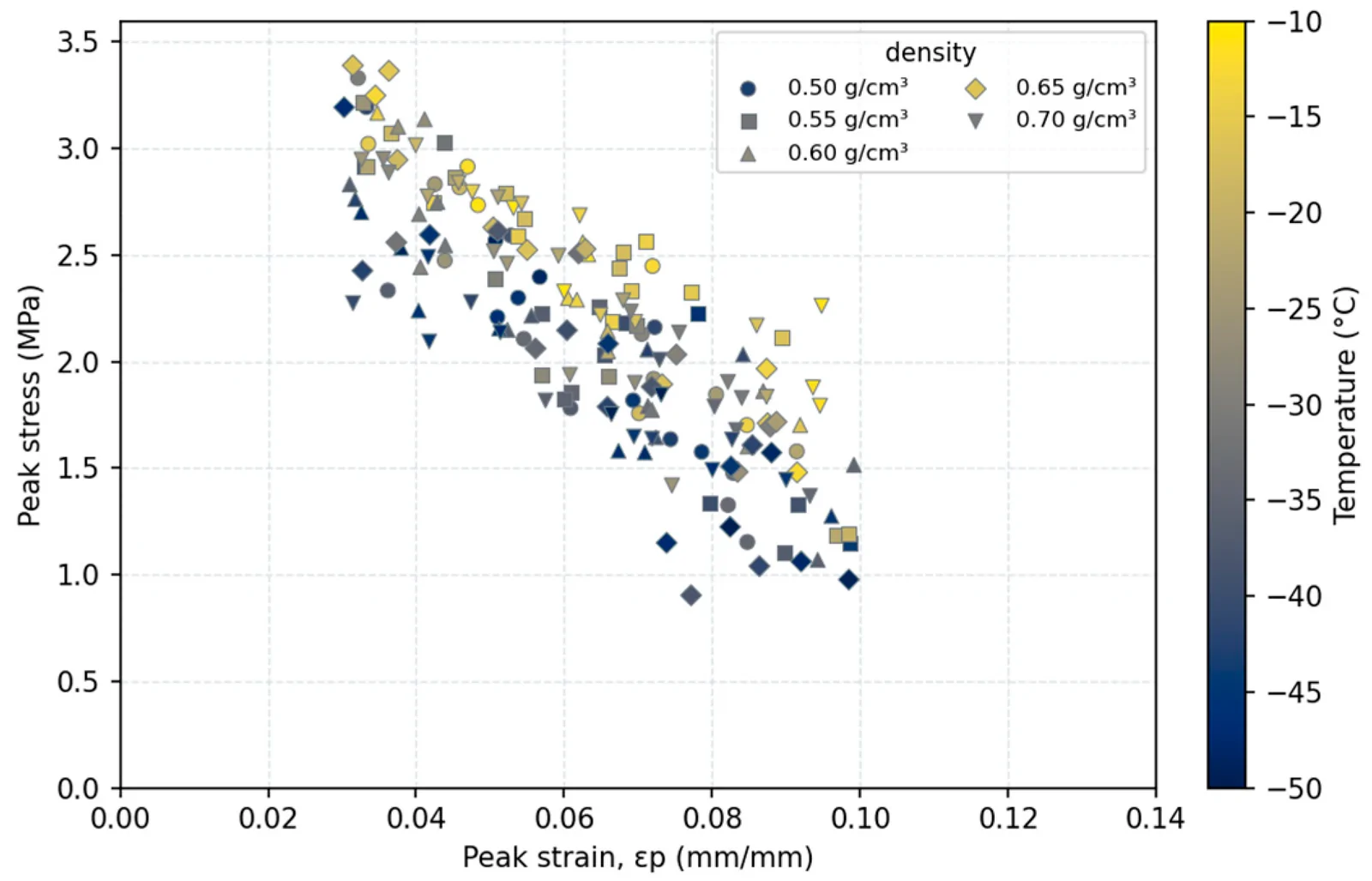
Peak strength and peak strain across the experimental conditions. Symbols distinguish density, and color denotes temperature in °C. Stress is in MPa, and strain is dimensionless.

Figure 8 shows that peak capacity and peak strain describe complementary mechanical properties. Similar peak strains occur at different strength levels because the amount of deformation does not uniquely specify the load carried by the contacts. The density series combines greater capacity with less rearrangement before the peak, whereas temperature changes the resistance to contact deformation. This distinction also explains why an overall strength-strain correlation can combine several physical effects. Schöttner et al. fitted a power-law density dependence to depth-hoar strength and then related the regression residuals to horizontal microstructural dimensions [21]. Their analysis separates the contribution of solid fraction from the geometry through which force is transmitted. Fourteau et al. likewise found different compaction behavior for depth hoar and rounded grains at similar density [50]. These findings support evaluating the present capacity and deformation measures together. Density describes the compacted state, while temperature and loading rate determine how that state responds during compression.

### 3.2. Mechanical and Electrical Responses Under Multiple Factors

#### 3.2.1. Temperature-Dependent Electrical Level and Mechanical Transition

**Figure 9:**
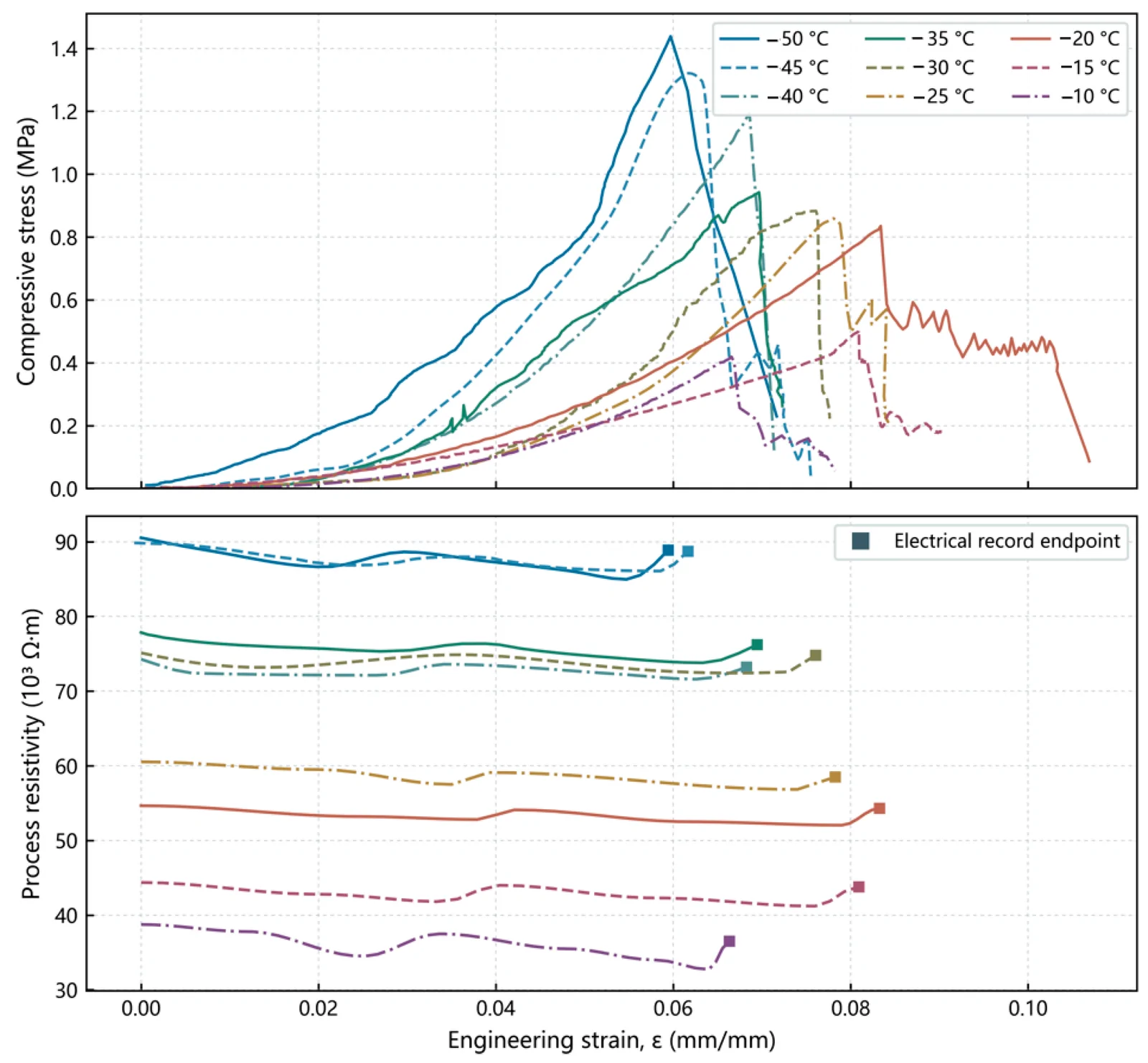
Mean stress and process apparent resistivity across temperature at 0.50 g/cm^3^ and 25 mm/min. Matching colors and line types identify the same condition. Squares denote electrical endpoints. Strain is dimensionless; stress is in MPa and apparent resistivity in 10^3^ Ω·m.

Figure 9 compares the mechanical and electrical responses recorded during compression at different temperatures. Cooling generally increased peak stress and initial apparent resistivity, but the two quantities changed by different proportions. During early compression, stress increased while apparent resistivity decreased. Contact development provides a common structural explanation, as it can improve load transfer while changing the geometry available for electrical transport. As mechanical softening began, apparent resistivity could continue to decline or remain nearly constant. This difference is consistent with the loss of critical load-bearing contacts occurring alongside continued contact adjustment elsewhere. The electrical response therefore provides complementary information on structural evolution after the onset of mechanical softening.

Among the temperature curves, the −20 °C response exhibited the most sustained post-peak stress fluctuations, with repeated stress drops and partial recoveries during continued compression. In contrast, the −50 and −45 °C curves showed more abrupt post-peak declines with less sustained recovery. This irregular macroscopic response at −20 °C is consistent with intermittent contact failure and subsequent load redistribution, allowing partial recovery of load-bearing capacity as deformation proceeded.

Temperature influences both charge transport and the polarization response of ice. Glen and Paren related the electrical properties of snow and ice to temperature, frequency, and phase arrangement [39]. At the excitation frequency of 50 kHz used here, the higher initial apparent resistivity under colder conditions is consistent with these temperature-dependent processes. During compression at a fixed temperature, increasing contact area and reducing intergranular gaps can lower the measured electrical resistance. Local contact separation or replacement can partially reverse this decrease. The resulting apparent resistivity reflects structural adjustment together with the electrical response of the contact interfaces, including those between the electrodes and snow. This interpretation links the broad resistivity decrease to compaction and the subsequent fluctuations to changes in the measured electrical pathways.

**Figure 10:**
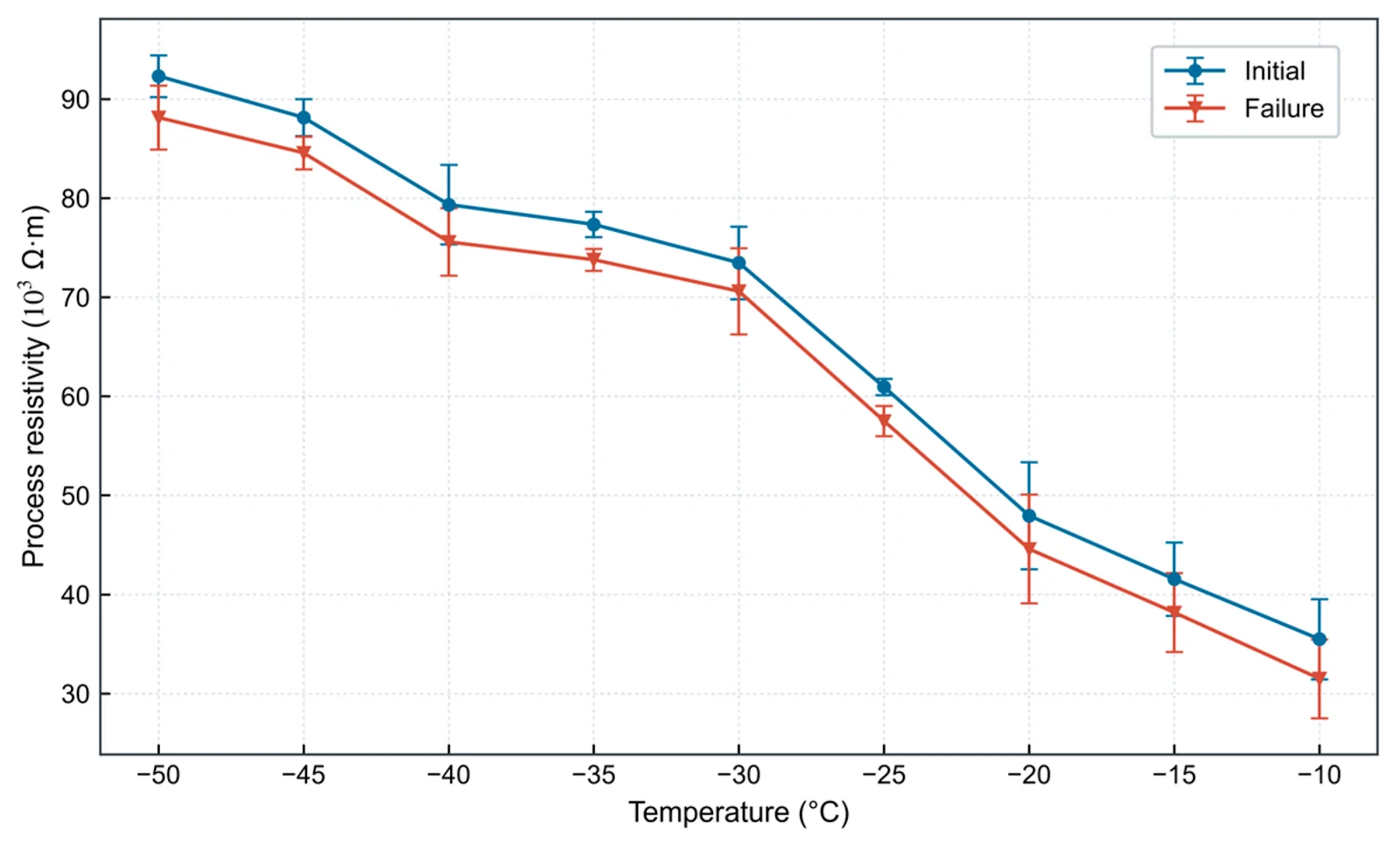
Initial, minimum, and terminal apparent resistivity across temperature under the conditions of Figure 9. The shaded region spans the initial and minimum values. Separate markers denote the three electrical values. Apparent resistivity is in 10^3^ Ω·m.

Figure 10 distinguishes the initial electrical level from the relative response to compression. Initial apparent resistivity decreased from 90.538 × 10^3^ Ω·m at −50 °C to 38.747 × 10^3^ Ω·m at −10 °C. In contrast, the maximum relative decrease during loading increased from 6.16% to 15.53%, approximately 2.521-fold. The warmer snow therefore underwent a larger proportional electrical change despite its lower initial resistivity. Thermally facilitated contact deformation can increase the scope for contact-area adjustment during loading, while temperature-dependent transport sets the initial electrical level [28,39]. These two contributions explain why absolute resistivity and relative resistivity change carry different information. Normalization by the initial value separates the scale of the electrical response from the baseline temperature effect.

The stress peak and resistivity minimum mark different balances within the evolving contact structure. Peak stress occurs when additional compression no longer compensates for loss of mechanical capacity. The electrical minimum occurs when the net decrease in apparent resistivity ceases. Their separation is physically meaningful because a contact that carries little axial force can still contribute to the measured electrical path. Conversely, failure of a highly loaded contact can reduce stress before other paths stop improving. The simultaneous bond rupture and contact creation described by Hagenmuller et al. provide a structural basis for this interpretation [16]. Comparing the two feature strains therefore connects the onset of net load loss to continued contact adjustment.

#### 3.2.2. Density-Driven Load Capacity and Transition Shift

**Figure 11:**
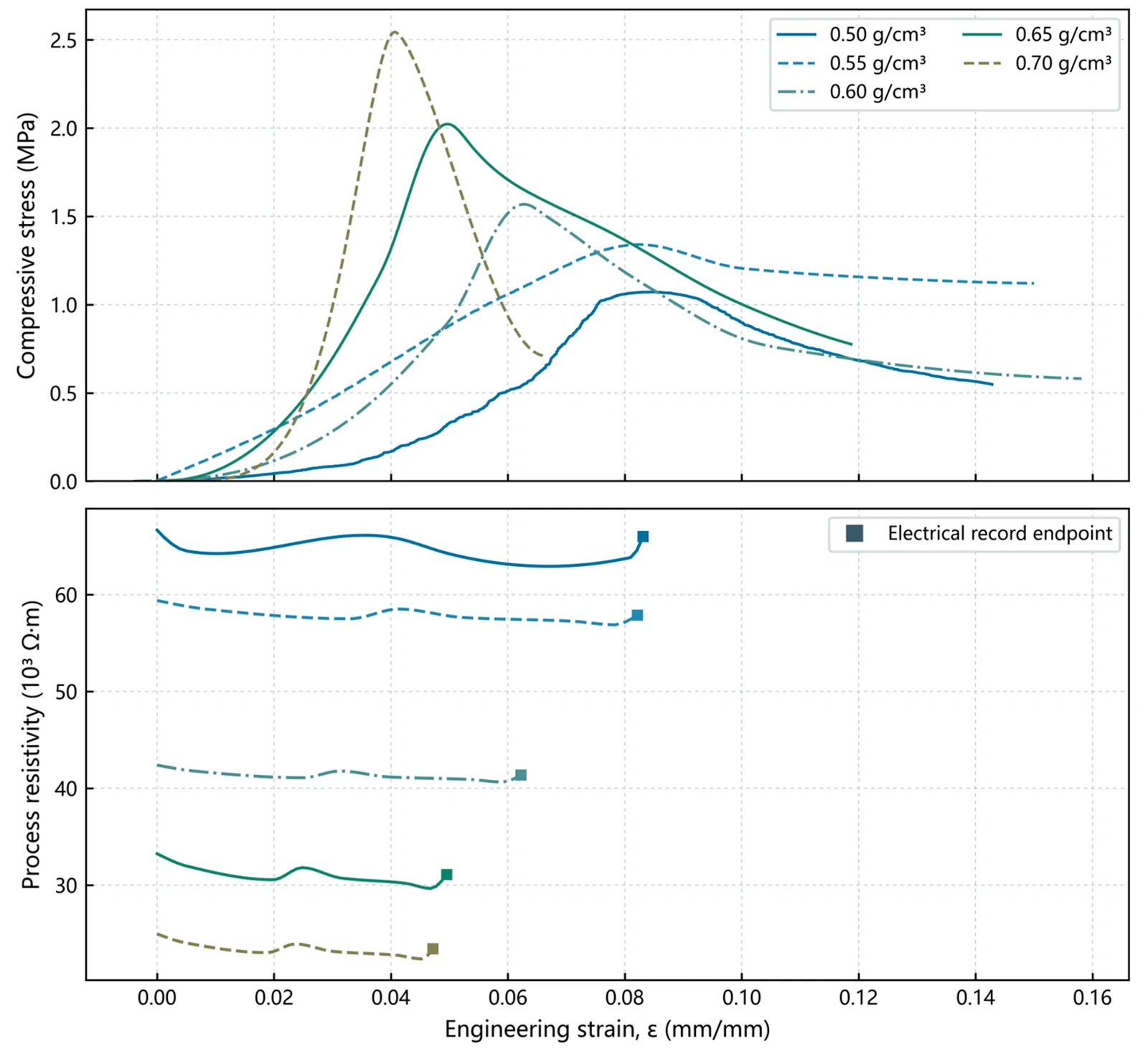
Mean stress and process apparent resistivity across density at −30 °C and 15 mm/min. Squares denote electrical endpoints. Strain is dimensionless; stress is in MPa and apparent resistivity in 10^3^ Ω·m.

Figure 11 shows that densification strengthens the mechanical response while lowering the initial electrical level. From 0.50 to 0.70 g/cm^3^, peak strength rose to approximately 2.373 times its initial value and initial apparent resistivity decreased from 66.652 × 10^3^ to 24.953 × 10^3^ Ω·m, a reduction of 62.6%. More effective contacts can distribute force through a larger connected ice skeleton and provide additional routes for electrical transport. Reduced pore space also limits deformation by rearrangement, bringing the stress peak to a lower strain. This interpretation is consistent with compression imaging and contact-based modeling [15,16], together with the role of phase arrangement in snow electrical properties [39]. Cooling, by comparison, raised both strength and initial resistivity. The contrasting directions distinguish structural strengthening through densification from temperature-dependent changes in ice mechanics and electrical transport. This distinction makes the paired measurements more informative than an absolute resistivity value considered alone.

The post-peak portions of Figure 11 show that loss of mechanical capacity can coexist with continued electrical improvement. When a critical load-bearing contact fails, its force is transferred to neighboring contacts and may promote further local failure. Compression can nevertheless close pores and create contacts in less highly stressed regions. Those contacts may contribute to electrical transport without restoring the previous peak load. Hagenmuller et al. incorporated bond rupture, contact creation, and grain rearrangement within the same compression model [16]. Their coexistence explains how resistivity can remain nearly constant or continue to decrease after stress has started to fall.

**Figure 12:**
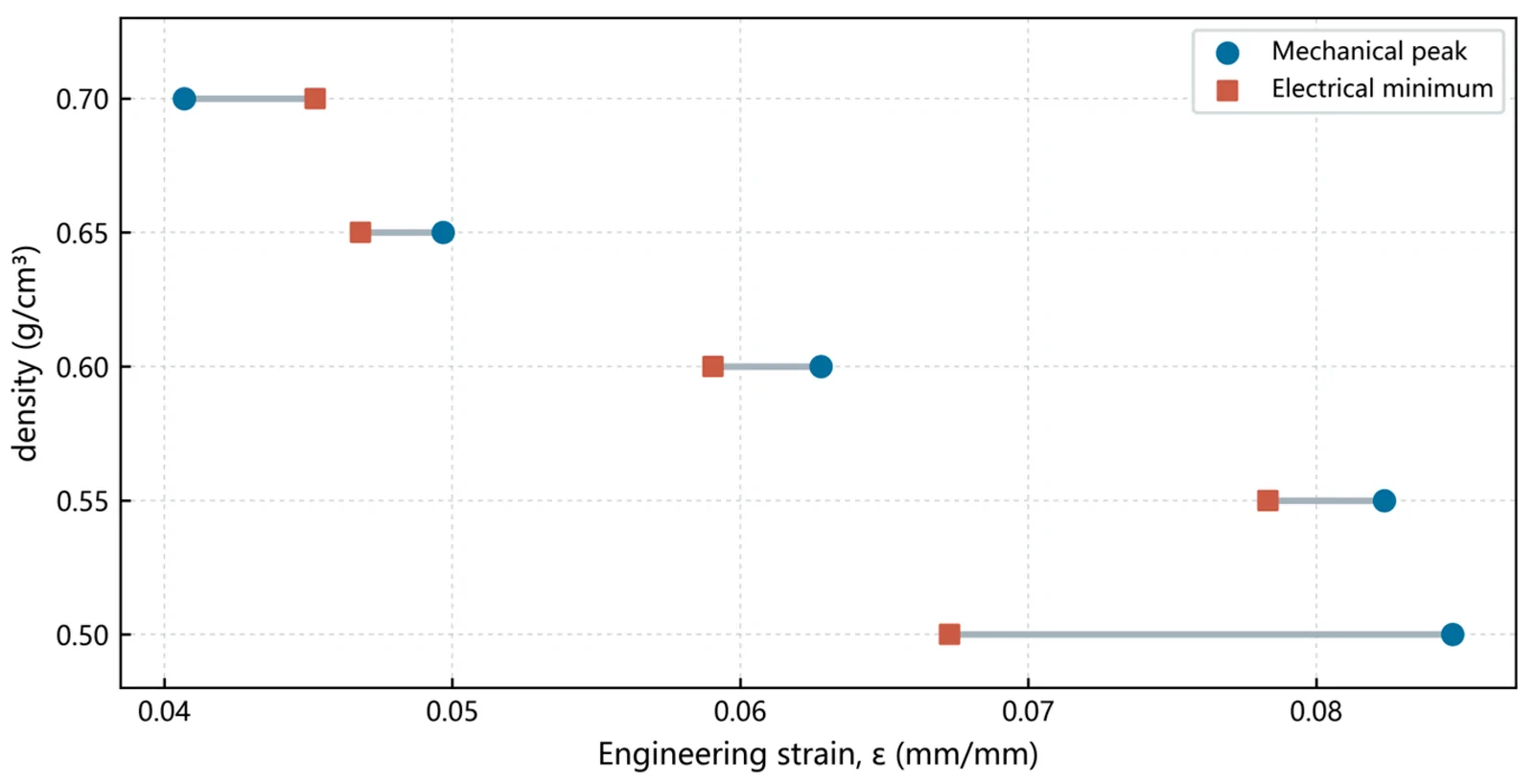
Strain positions of peak stress and minimum apparent resistivity across density under the conditions of Figure 11. Horizontal lines connect the mechanical peak and electrical minimum. Circles denote stress peaks, and squares denote resistivity minima. Strain is dimensionless.

Figure 12 shows that both the magnitude and direction of the feature separation change with density. The separation is expressed as $\Delta \varepsilon \;=\;\varepsilon_{\rho_{\min}}\;-\;\varepsilon_{p}$. A positive value places the resistivity minimum after the stress peak and identifies continued net electrical improvement during mechanical softening. A negative value indicates that the electrical trajectory has already turned upward while stress is still increasing. These two sequences are compatible with different mechanical and electrical weights of individual contacts. Highly loaded bonds govern the peak capacity, whereas the electrical response combines contributions from paths with different orientations and contact geometries [16,39]. The change in sign therefore provides information about the sequence of contact reorganization that is not captured by peak strength alone.

The density-dependent feature separation motivates distinct mechanical and electrical references in the constitutive description. The stress peak fixes the capacity from which subsequent load loss is measured. The electrical reference follows the lowest resistivity reached during further loading. This arrangement preserves the observed sequence when contact failure and contact creation occur together [16]. It also connects the macroscopic softening response to an electrical history that can continue evolving after the mechanical peak.

#### 3.2.3. Loading-Rate Effects on Resistivity Rebound and Feature Separation

**Figure 13:**
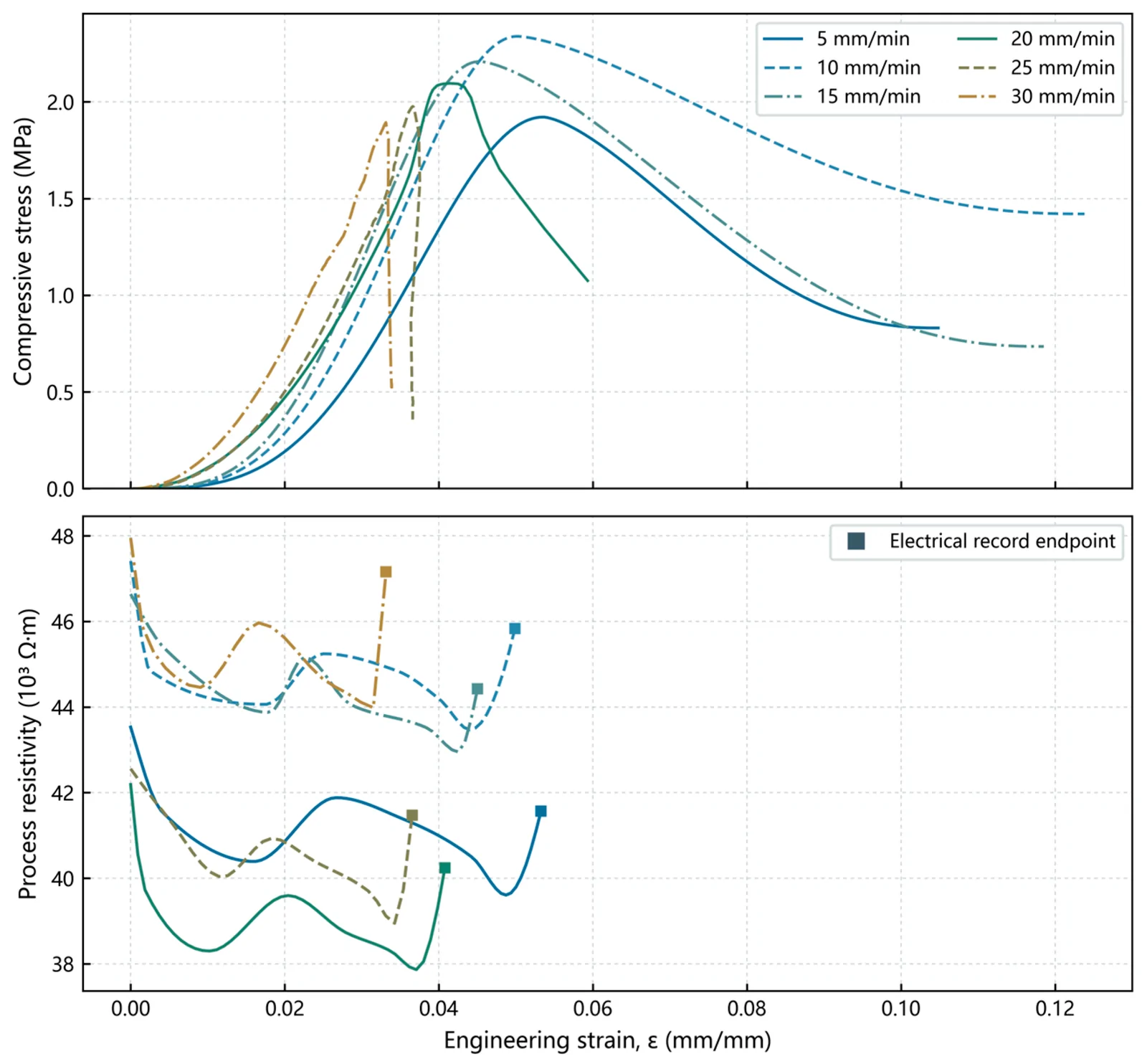
Mean stress and process apparent resistivity across loading rate at 0.65 g/cm^3^ and −40 °C. Squares denote electrical endpoints. Strain is dimensionless; stress is in MPa and apparent resistivity in 10^3^ Ω·m.

Figure 13 shows that loading rate alters the strain interval of the mechanical and electrical transitions. At 0.65 g/cm^3^ and −40 °C, faster loading shifted the stress peak to lower strain, while strength was highest at 10 mm/min. Initial apparent resistivity ranged from 42.190 × 10^3^ to 47.952 × 10^3^ Ω·m without a monotonic ordering. The clearer rate dependence emerged during loading, through the positions of the electrical minima and the later rebound. This distinction directs attention to the evolution of contacts under compression rather than to differences in their initial electrical level.

Loading rate changes the competition between stress accumulation and structural adjustment. At slower rates, viscoplastic deformation can release stress concentrations and allow neighboring contacts to redistribute the load. At higher rates, stress can accumulate before these adjustments are completed, favoring earlier mechanical instability [12]. Reiweger et al. represented rate-dependent failure through competing rupture and rebonding timescales [44], and Xiao et al. extended this picture to compacted-snow compression [46]. Within this interpretation, a later resistivity rebound records the electrical consequence of contact separation and reorganization. The paired trajectories thus reveal how a change in loading duration affects capacity and the subsequent contact configuration.

**Figure 14:**
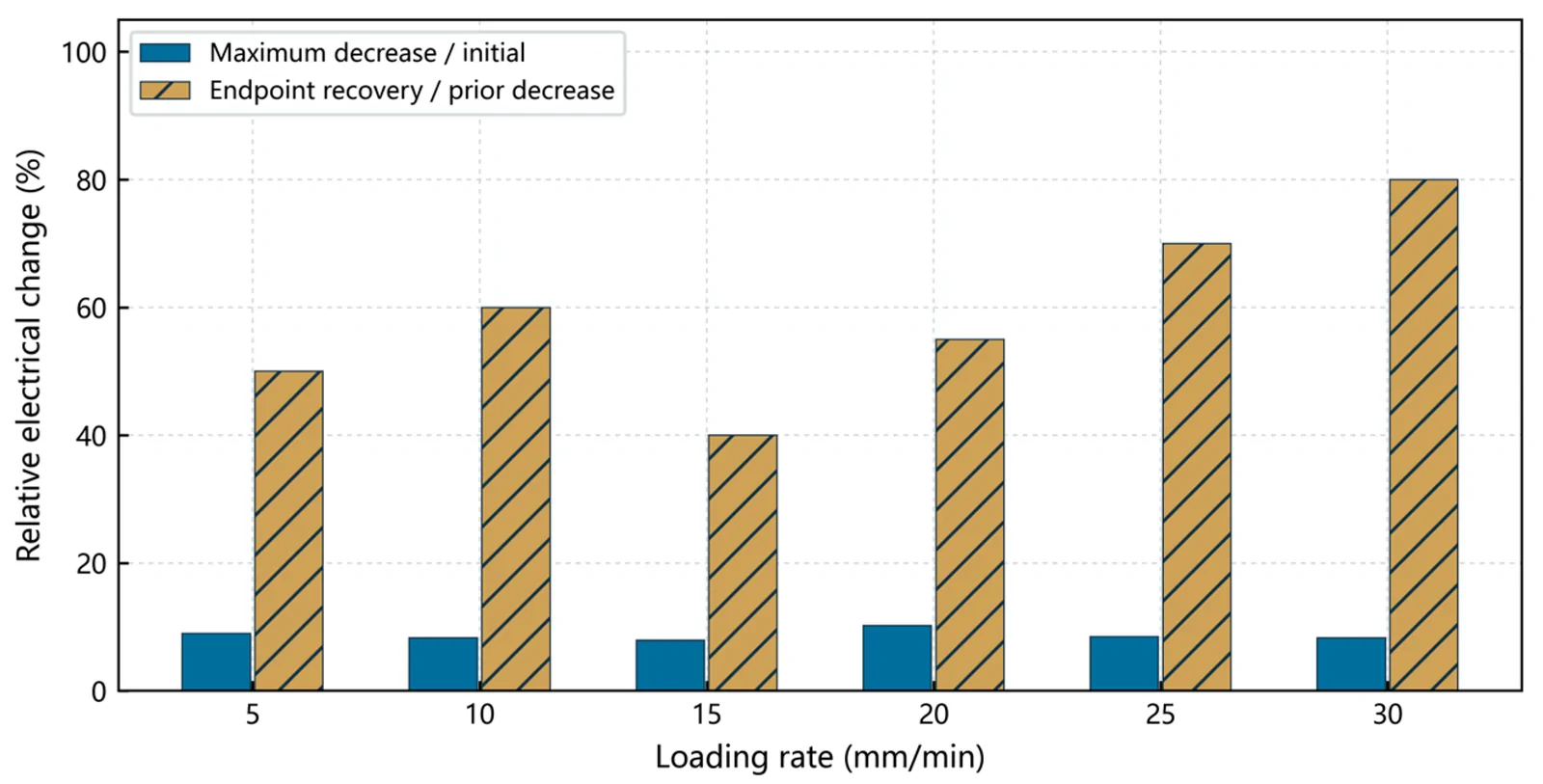
Maximum relative resistivity decrease and terminal rebound ratio across loading rate under the conditions of Figure 13. The bars give 100Aρ and 100Bρ in percent. The terminal ratio is evaluated at the electrical endpoint marked in Figure 13.

Figure 14 shows a stronger rate dependence of the late electrical rebound than of the maximum relative resistivity decrease. The decrease ranged from 7.91% to 10.25%, a span of 2.34 percentage points. The terminal rebound ratio rose from approximately 40% to 80% between 15 and 30 mm/min. The two amplitudes are defined by $A_{\rho }\;=\;\frac{\rho_{0}\;-\;\rho_{\min}}{\rho_{0}}$ and $B_{\rho }\;=\;\frac{\rho_{\mathrm{end}}\;-\;\rho_{\min}}{\rho_{0}\;-\;\rho_{\min}}$, with positive denominators; multiplication by 100 gives percentages. The first measures electrical improvement relative to the initial state, while the second measures how much of that improvement has been reversed at the endpoint. Their different trends suggest that contact disruption after the minimum is more rate-sensitive than the preceding net electrical decrease over these loading paths. Faster loading can limit the redistribution that compensates for lost contacts, leaving a larger electrical rebound. Figure 13 provides the corresponding strain positions, and Figure 14 summarizes the change in amplitude. Together, they distinguish earlier attainment of a feature from a larger subsequent electrical change.

### 3.3. Local Evaluation of Peak-Referenced Post-Peak Softening

#### 3.3.1. Mechanical and Electrical State Descriptors

The mechanical and electrical results motivate two complementary state descriptions. The stress peak establishes the reference for subsequent mechanical softening. Apparent resistivity records the net electrical change as contacts develop, separate, and reorganize. Because continued compaction can coexist with load loss, the electrical reference follows its own trajectory. The relative decrease from the initial electrical state is defined as:

(3) $$S_{\rho }(\varepsilon )=\max\left[0,\frac{\rho_{0}-\rho (\varepsilon )}{\rho_{0}}\right]$$

Here, *S_ρ_* is the dimensionless positive relative decrease in process apparent resistivity, *ρ*_0_ is its initial value, and *ρ*(*ε*) is its value at strain ε. For positive resistivity, 0 ≤ *S*_*ρ*_ < 1. A larger *S*_*ρ*_ indicates a greater reduction from the initial electrical level. It is useful for comparing the compaction-related electrical response across different baselines. When resistivity rises, *S*_*ρ*_ decreases and records the reversal of part of that electrical change.

After the stress peak, continued pore closure can still reduce resistivity while mechanical capacity declines. A running minimum retains this evolving electrical reference:

(4) $$\rho_{l}(\varepsilon )=\min_{\varepsilon_{p}\leq \xi \leq \varepsilon }\rho (\xi ),\;\varepsilon \;\geq \;\varepsilon_{p}$$

Here, *ρ*_l_ is the lowest apparent resistivity reached between the peak strain ε_p_ and the current strain, and ξ denotes historical strain. Equation (4) updates the reference whenever compression produces a lower electrical value. The reference can therefore continue changing after mechanical softening has started. The subsequent logarithmic rise is measured relative to this running minimum:

(5) $$Q_{\rho }(\varepsilon )=\max_{\varepsilon_{p}\leq \eta \leq \varepsilon }\ln\left[\frac{\rho (\eta )}{\rho_{l}(\eta )}\right],\;\varepsilon \;\geq \;\varepsilon_{p}$$

Here, *Q*_*ρ*_ is a dimensionless electrical history variable, and *η* denotes historical strain. At the peak, *Q*_*ρ*_ = 0 and the running minimum is initialized with the resistivity at *ε*_p_. The discrete updates are *ρ*_*l*,*i*_ = min(*ρ*_*l*,*i*−1_,*ρ_i_*) followed by $Q_{i}=\max\left(Q_{i-1},\ln\frac{\rho_{i}}{\rho_{l,i}}\right)$, using positive resistivity values. Equation (5) retains the largest logarithmic rise above the running minimum. This history rule preserves an established rebound when a later fluctuation temporarily reduces resistivity. In contrast, *S_ρ_* follows the current decrease from the initial state. The two variables therefore separate the instantaneous relative electrical change from the largest post-peak rebound, providing distinct inputs for the subsequent mechanical comparison.

**Figure 15:**
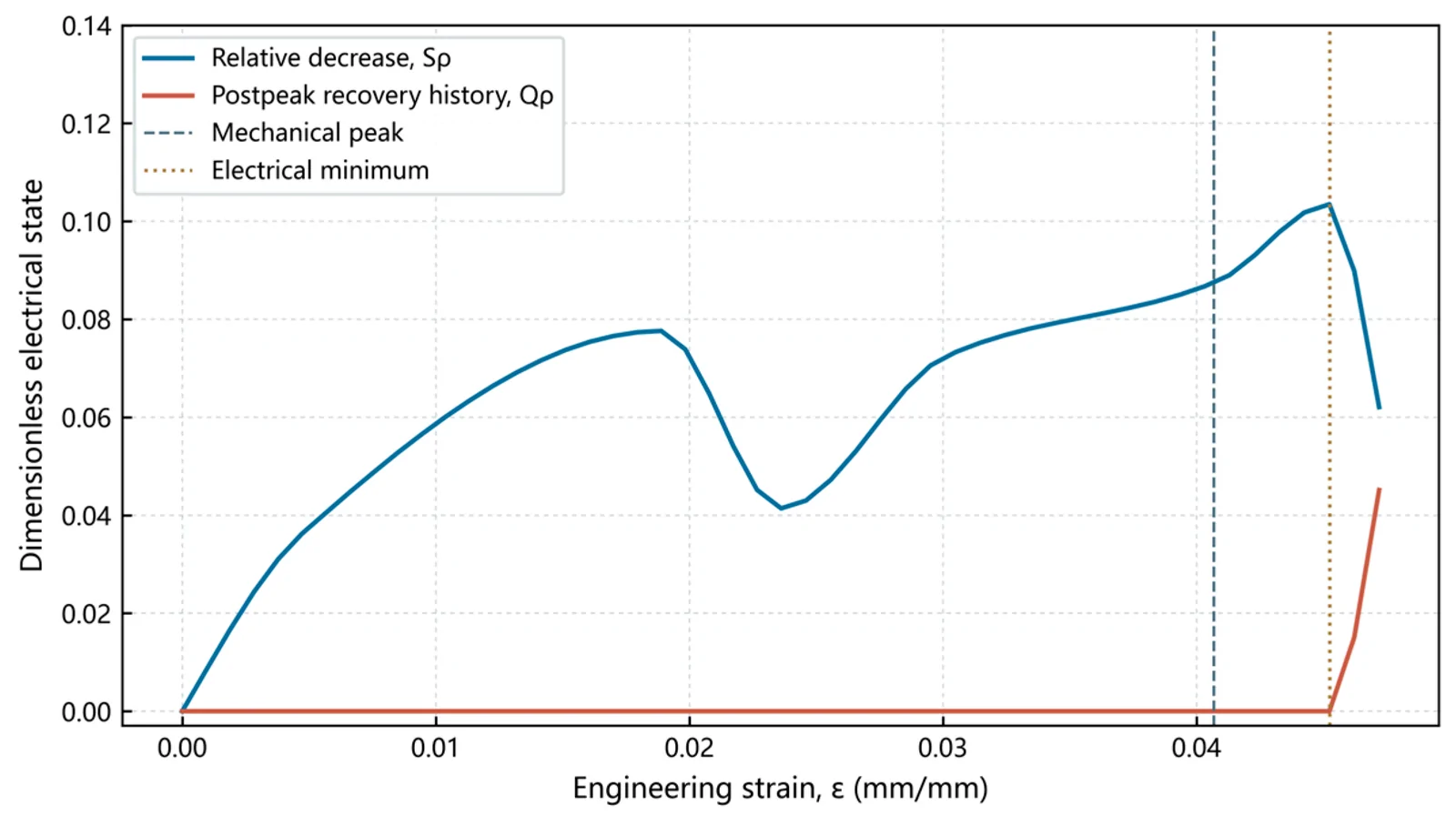
Relative resistivity decrease S_*ρ*_ and post-peak rebound history Q_*ρ*_ at 0.70 g/cm^3^, −30 °C, and 15 mm/min. Vertical lines denote the stress peak and resistivity minimum. Both variables and strain are dimensionless. Q_*ρ*_ uses the post-peak running minimum as its reference.

In Figure 15, S_*ρ*_ is already elevated near peak stress, whereas Q_*ρ*_ increases only after resistivity rises above its post-peak minimum. This sequence indicates that mechanical softening begins before the electrical rebound becomes established. Continued adjustment in less highly loaded contacts offers an explanation for the intervening decrease in resistivity. Contact-based simulations describe the coexistence of rearrangement and bond rupture during compression [16,35]. The present state variables translate that sequence into quantities that can be compared with the stress loss. Section 3.3.3 then evaluates the contribution of rebound history after accounting for strain-dependent softening.

#### 3.3.2. Peak-Referenced Weibull Baseline

The model separates reference-stress growth during continued deformation from the loss of an equivalent load-bearing fraction. It uses the one-dimensional effective-stress relation:

(6) $$\sigma =(1-D)\tilde{\sigma },\;\tilde{\sigma }=E_{0}\varepsilon$$

Here, $\tilde{\sigma }$ is the equivalent stress of the reference structure, *D* is its equivalent damage, and *E*_0_ is a reference stiffness scale. Equation (6) follows the effective-stress concept [42]. It separates the stress sustained by the reference structure from the fraction of capacity retained during loading. *E*_0_ is fixed by the peak constraint and represents the load-bearing scale of the compacted structure near the peak. The linear reference is used to describe subsequent softening, while the earlier compaction stage establishes the structure to which that reference applies.

Variations in bond geometry and orientation produce different local force levels under the same macroscopic strain. Failure can therefore develop progressively across load-bearing regions instead of occurring everywhere at once. A Weibull distribution represents this dispersion through equivalent failure-strain thresholds [43]. Snow fiber-bundle models use distributed strengths to describe progressive failure [44], and recent compression models also account for strengthening and rebonding [46]. Here, the peak-referenced baseline summarizes the net loss of capacity after these competing processes have produced the stress maximum. The equivalent threshold *ε*_t_ is written as:

(7) $$P(\varepsilon_{t}\leq \varepsilon )=D_{W}(\varepsilon )=1-\exp\left[-{\left(\frac{\varepsilon }{\varepsilon_{0}}\right)}^{m}\right],\;m>0,\;\varepsilon_{0}>0$$

Here, *P* is the cumulative failure probability, *D_W_* is equivalent statistical damage, m controls the threshold distribution, and *ε*_0_ is its strain scale. At fixed *ε*_0_, increasing m concentrates failure thresholds around that scale. The remaining fraction 1 − DW describes the part of the reference capacity still contributing to stress. This statistical representation connects progressive local failure to a smooth macroscopic softening curve. Substitution into Equation (6) gives:

(8) $$\sigma_{W}(\varepsilon )=E_{0}\varepsilon \exp\left[-{\left(\frac{\varepsilon }{\varepsilon_{0}}\right)}^{m}\right]$$

Here, *σ_W_* is the stress calculated from the Weibull baseline. Equation (8) balances the growth of reference stress with the decline of the remaining load-bearing fraction. Its logarithmic derivative is $\frac{d\ln\sigma_{W}}{d\varepsilon }=\frac{1}{\varepsilon }-\frac{m\varepsilon^{m-1}}{\varepsilon_{0}^{m}}$ for positive strain. The first term describes reference-stress growth, while the second describes the increasing contribution of degradation. At the peak these contributions are equal. Their balance yields:

(9) $$\varepsilon_{p}=\varepsilon_{0}m^{-\frac{1}{m}},\;\sigma_{p}=E_{0}\varepsilon_{p}e^{\left(-\frac{1}{m}\right)}$$

Here, σ_p_ and ε_p_ are the peak stress and peak strain. Equation (9) links the observed transition from hardening to softening to the statistical strain scale. Applying both peak constraints gives:

(10) $$\varepsilon_{0}=\varepsilon_{p}m^{\frac{1}{m}},\;E_{0}=\left(\frac{\sigma_{p}}{\varepsilon_{p}}\right)e^{\left(\frac{1}{m}\right)}$$

Equation (10) determines *E*_0_ and *ε*_0_ from the peak, leaving m to govern the post-peak shape. This parameter reduction ties the regression to the measured load maximum rather than allowing an arbitrary stress or strain shift. Contact adjustment before the peak is incorporated into that reference state. Additional degradation is then defined relative to the capacity remaining at the peak:

(11) $$D_{W}^{*}(\varepsilon )=1-\frac{1-D_{W}(\varepsilon )}{1-D_{W}(\varepsilon_{p})}=1-\exp\left\{-\frac{{\left(\frac{\varepsilon }{\varepsilon_{p}}\right)}^{m}-1}{m}\right\}$$

**Figure 16:**
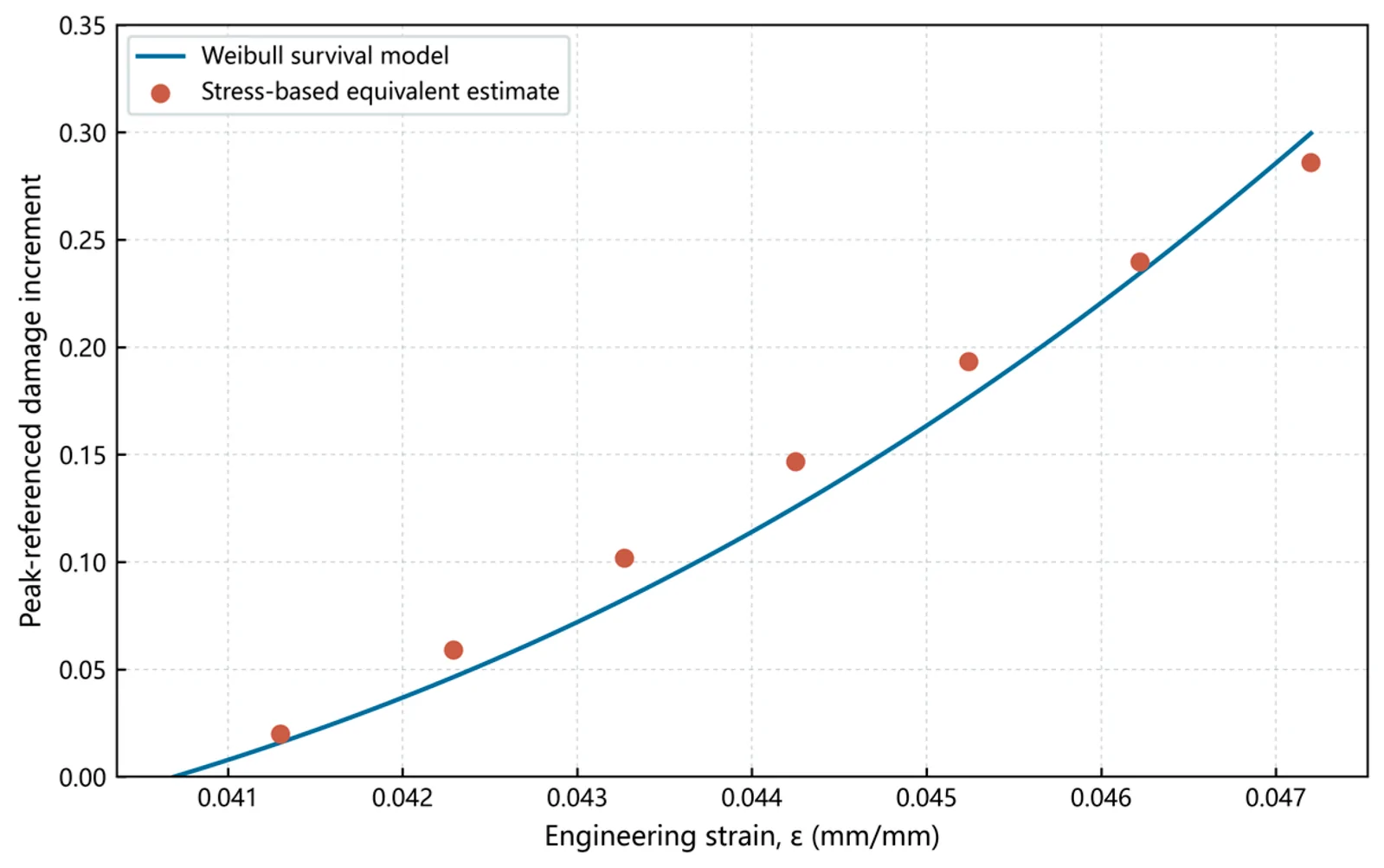
Peak-referenced equivalent damage under the conditions of Figure 15. Points give 1 − σε_p_/(σ_p_ε), and the line gives the fitted Weibull expression in Equation (11).

Here, $D_{W}^{*}$ is the additional equivalent damage relative to the peak-surviving capacity. With *D_p_* = *D_W_*(*ε_p_*), the same normalization is $D_{W}^{*}=\frac{D_{W}-D_{p}}{D_{p}}$. The subtraction isolates the post-peak increase in equivalent damage, and the denominator expresses that increase relative to the fraction remaining at the peak. Consequently, *D*_W_* starts from zero and increases as this remaining fraction decreases. Figure 16 compares the Weibull expression with 1 − σ*ε*_p_/(*σ*_p_ε), the equivalent quantity derived from stress. Their comparison tests how the fitted softening shape represents the normalized capacity loss. *Q_ρ_*, by contrast, records the electrical rebound. The two descriptions retain the different mechanical and electrical sequences identified in Section 3.2.

#### 3.3.3. Test of an Incremental Electrical Term

**Figure 17:**
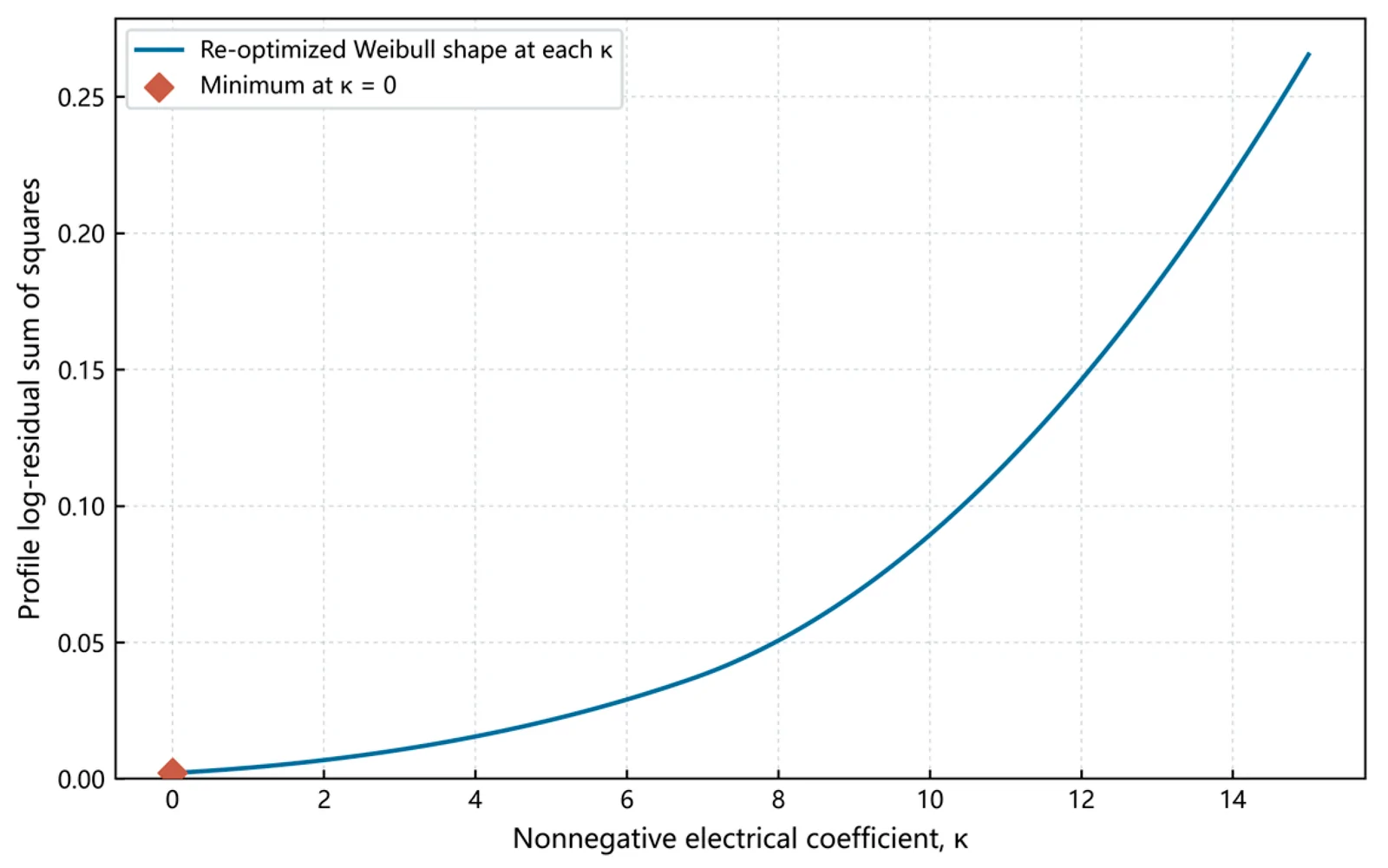
Profile of the regression objective for nonnegative κ under the conditions of Figure 15. The parameter m is reoptimized at each κ. The ordinate gives the squared logarithmic residual sum from Equation (15), with a minimum at *κ* = 0.

Figure 17 evaluates the electrical term within a nested nonlinear regression. The strain-dependent baseline is retained, and rebound history supplies a candidate additional contribution to capacity loss. This construction tests whether the electrical change accounts for stress variation beyond that already associated with increasing strain. The additional cumulative hazard is written as:

*H_ρ_* = *κQ_ρ_*, *D_R_* = 1 − exp(−*H_ρ_*), *κ* ≥ 0 (12)

Here, *H_ρ_* is the additional model hazard associated with resistivity rebound, *κ* is its dimensionless coefficient, and *D_R_* is the corresponding damage term. The nonnegative constraint makes this term represent additional attenuation of load capacity. Setting κ = 0 recovers the strain-dependent baseline, so the two descriptions can be compared within the same mechanical framework [42,44]. The remaining capacities are combined multiplicatively:

(13) $$D^{*}=1-(1-D_{W}^{*})(1-D_{R})$$

Here, D* is the combined peak-referenced damage. Equation (13) applies the electrical attenuation to the capacity remaining after the strain-dependent contribution. Multiplication keeps the remaining fraction between zero and one and makes the two model hazards additive in logarithmic form. Conversion to the original reference in Equation (6) gives 1 − *D*_tot_ = (1 − *D_p_*)(1 − D^*^), followed by $\sigma =E_{0}\varepsilon \left(1-D_{p}\right)\left(1-D^{*}\right)=\frac{\sigma_{p}}{\varepsilon_{p}}\varepsilon \left(1-D^{*}\right)$. *D*_tot_ refers to the original reference structure, whereas D* refers to the structure at peak stress. This distinction preserves the peak load while representing the additional post-peak decline. Substitution yields:

(14) $$\sigma (\varepsilon )=\sigma_{p}\left(\frac{\varepsilon }{\varepsilon_{p}}\right)\exp\left\{-\frac{{\left(\varepsilon /\varepsilon_{p}\right)}^{m}-1}{m}-\kappa Q_{\rho }(\varepsilon )\right\},\;\varepsilon \geq \varepsilon_{p}$$

Equation (14) applies for *ε* ≥ *ε*_p_. The peak stress and peak strain fix the reference scales, *m* controls the curvature of strain-dependent softening, and *κ* controls the additional attenuation associated with rebound history. At the peak, Qρ = 0 and the relation gives *σ* = *σ_p_*. The baseline has a horizontal peak tangent. For a differentiable electrical history, that tangent is retained when κQ′ρ(*ε*_p_+) = 0. The formulation therefore accommodates the measured separation of the mechanical peak and electrical minimum while maintaining an explicit mechanical reference.

The parameters are estimated by nonlinear least squares on logarithmic stress ratios. This transformation expresses deviations relative to the local stress level and compares the baseline and electrical extension on the same scale:

(15) $$J(m,\kappa )=\sum_{i}{\left\{\ln\left[\frac{\sigma_{i}}{\sigma_{p}\varepsilon_{i}/\varepsilon_{p}}\right]+\frac{{\left(\varepsilon_{i}/\varepsilon_{p}\right)}^{m}-1}{m}+\kappa Q_{i}\right\}}^{2}$$

Here, *σ*_i_ is stress at strain *ε*_i_ and *Q*_i_ is the corresponding electrical history. With x = ε/*ε*_p_, the regression can be written as $-\ln\left[\frac{\sigma }{\sigma_{p}x}\right]\;=\;\frac{x^{m}\;-\;1}{m}\;+\;\kappa Q_{\rho }$. The normalized stress loss is thus separated into a nonlinear strain term and a term linear in κ. Equation (15) gives equal weight to squared logarithmic residuals. In Figure 17, m is reoptimized at each fixed κ, and the objective increases from its minimum at κ = 0. The regression therefore selects the strain-dependent baseline for this interval. The sensitivity $\frac{\partial \ln\sigma }{\partial \kappa }=-Q_{p}$ explains the role of the electrical term: it lowers calculated stress only where *Q_ρ_* is positive. Here, this direct effect occurs at the final two positions, after softening is already established.

#### 3.3.4. Local Calibration and Parameter Sensitivity

**Figure 18:**
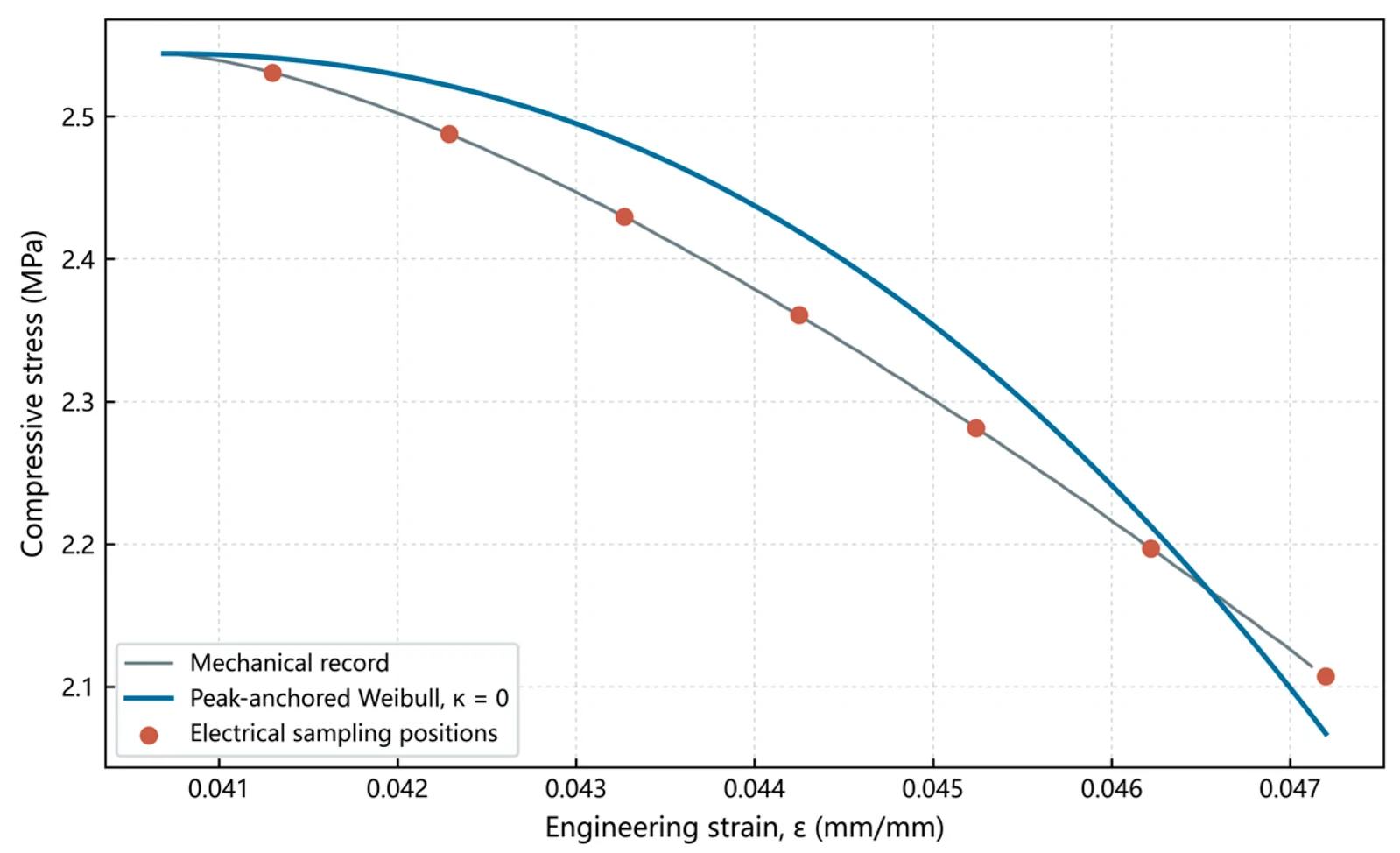
Local nonlinear regression of the post-peak stress response under the conditions of Figure 15. The gray line gives the mechanical response, points denote the seven electrical sampling strains, and the blue line gives Equation (17) over the fitted interval.

Figure 18 compares the fitted relation with the compression response at 0.70 g/cm^3^, −30 °C, and 15 mm/min. The regression follows the stress decline after the peak while preserving the measured peak scales. With *m* fixed, the residual is defined as the natural logarithm of the ratio of measured stress to the stress calculated by the model without the electrical term. Estimating κ is then a constrained linear projection of these residuals onto the electrical history. Minimization gives:

(16) $$\kappa^{*}(m)=\max\left[0,-\frac{\sum_{i}Q_{i}a_{i}}{\sum_{i}Q_{i}^{2}}\right]$$

Here, κ* is the optimal nonnegative electrical coefficient for a specified m. With Σ$Q_{i}^{2}$ > 0, Equation (16) reduces the joint fit to a one-dimensional search in *m*. A logarithmic search over 0.01 ≤ *m* ≤ 150 followed by interval refinement gave *m* = 10.463 and *κ* = 0. The peak scales are σ_p_ = 2.544 MPa and ε_p_ = 4.069 × 10^−2^, with E_0_ = 68.794 MPa and ε_0_ = 5.093 × 10^−2^. This peak constraint gives the shape parameter a direct mechanical interpretation. For *x* = ε/*ε*_p_ and κ = 0, ${\left.\frac{d^{2}\left(\sigma /\sigma_{p}\right)}{dx^{2}}\right|}_{x=1}=-m$. The fitted m therefore sets how rapidly the response curves downward from the peak. The numerical relation is:

(17) $$\sigma (\varepsilon )=2.544\left(\frac{\varepsilon }{4.069\times 10^{-2}}\right)\exp\left[-\frac{{\left(\frac{\varepsilon }{4.069\times 10^{-2}}\right)}^{10.463}-1}{10.463}\right]$$

In Equation (17), *σ* is in MPa and *ε* is dimensionless. The fitted interval extends from ε = 4.069 × 10^−2^ to 4.720 × 10^−2^. At its upper end, x is approximately 1.160 and the model gives σ/*σ*_p_ = 0.813 and *D*_W_^*^ = 0.300. The equivalent capacity loss exceeds the relative stress decrease because the reference stress grows with continued strain. This distinction explains why stress reduction alone does not equal the peak-referenced damage variable. The selected κ = 0 retains the mechanical baseline, while the electrical variables describe the accompanying contact adjustment and rebound.

Fit quality is evaluated in the original stress units as well as through the logarithmic regression objective:

(18) $$\mathrm{RMSE}=\sqrt{\frac{1}{N_{\varepsilon }}\sum_{i=1}^{N_{\varepsilon }}{\left({\hat{\sigma }}_{i}-\sigma_{i}\right)}^{2}},\;\;R^{2}=1-\frac{\sum_{i=1}^{N_{\varepsilon }}{\left({\hat{\sigma }}_{i}-\sigma_{i}\right)}^{2}}{\sum_{i=1}^{N_{\varepsilon }}{\left(\sigma_{i}-\bar{\sigma }\right)}^{2}}$$

Here, ${\hat{\sigma }}_{i}$ is calculated stress, $\bar{\sigma }$ is the average over the fitted positions, and N_ε_ = 7. Equation (18) gives RMSE = 0.041 MPa and R^2^ = 0.920. Relative to peak strength, the root-mean-square error is approximately 1.596%. Residuals defined as calculated minus observed stress are positive in the middle and negative near the end. The relation therefore slightly overestimates stress before underestimating the final retained capacity. This pattern directs attention to the evolution of softening curvature and the resistance carried by surviving or renewed contacts. Leaving out one position at a time changed m from 10.30 to 11.42, while κ remained zero. The stable selection of the baseline in this sensitivity check complements the objective profile in Figure 17. Together, the fit and residual analysis support a peak-referenced description of the local load decline.

The mechanical regression and electrical history describe complementary consequences of contact evolution. Stress depends strongly on the contacts that carry the largest forces, whereas apparent resistivity combines transport through the specimen with contact polarization [39]. Limbrock et al. found temperature-dependent conduction and polarization in partially frozen rocks over 10 mHz to 45 kHz [51]. This adjacent evidence supports treating the electrical contribution as frequency-dependent rather than as a direct count of failed contacts. In the present interval, the strain baseline describes the stress decline and the electrical variables retain the sequence of contact adjustment and rebound. The resulting framework connects the mechanical reference to a concurrent electrical observation without forcing the two feature strains to coincide. It provides a practical basis for interpreting load loss together with electrical change under the specified temperature and density conditions.

## 4. Conclusions

(1) The replicated factorial program showed different temperature, density, and loading-rate responses. At 0.50 g/cm^3^ and 25 mm/min, warming from −50 to −10 °C reduced reported peak strength by approximately 70.9%. At −30 °C and 15 mm/min, increasing density from 0.50 to 0.70 g/cm^3^ raised strength to approximately 2.37 times its initial value. Peak strain declined as density increased. The combination is consistent with improved load transfer and less space for rearrangement before the peak. Loading rate reduced peak strain while strength varied nonmonotonically. The reported within-condition CV summaries describe repeatability; adjusted factor contrasts require specimen-level analysis.
(2) Cooling and densification both increased strength, but their effects on initial process apparent resistivity had opposite signs. Densification lowered that electrical level by 62.6% in the reported density series. Electrical interpretation must therefore retain temperature and density as conditions. Peak stress and the observed resistivity minimum are distinct response features. Their strain separation provides the appropriate basis for testing correspondence within paired specimen records.
(3) The Weibull baseline described the reported local mean response over seven post-peak strain positions. The reported calibration gave m = 10.463, κ = 0, RMSE = 0.041 MPa, and R^2^ = 0.92. Only the final two positions had positive rebound history. The nonnegative electrical extension did not improve the fitted objective over that interval. The relation provides a local description of post-peak load loss and a test of additional electrical information. Its parameters describe the analyzed mean response and are not averages of specimen-specific fits. Xiao et al. [46] explicitly model densification and pressure sintering. Here, the Weibull relation summarizes the reported mean post-peak shape.
(4) The results provide a basis for interpreting concurrent measurements under specified material conditions. Field monitoring requires calibration for temperature, density, preparation history, and the electrode arrangement. Complete post-peak electrical records and independent batches are priorities for testing the local relation. Imaging or full-field deformation should examine the proposed contact mechanisms and their relation to the two observed signals. Future model development could represent compaction and evolving contacts explicitly.

## Figures and Tables

**Table 1:** Controlled factors and synchronized measurement settings.

Factor	Levels	Measurement
Temperature, T	−50 to −10 °C; 5 °C steps	Thermal conditioning and loading
Density, *ρ*_n_	0.50–0.70 g/cm^3^; 0.05 steps	Layered compaction; mass check
Loading rate, v	5–30 mm/min; 5 mm/min steps	Displacement control
LCR response, *ρ*(ε)	50 kHz; four-terminal	Synchronized record

## Data Availability

Replicate endpoint data, representative synchronized strain-indexed curves, and the seven-point local calibration dataset are available from the corresponding author upon reasonable request.

## References

[1] Abele G., Frankenstein G.E. (1967). Snow and Ice Properties as Related to Roads and Runways in Antarctica.

[2] Abele G. (1990). Snow Roads and Runways. CRREL Monograph 90-3.

[3] Duggan DiDominic K.L., Ordaz M., Melendy T.D., Charlebois C.M. (2025). Evaluating Snow Pavement Strength in Remote Cold Environments via California Bearing Ratio (CBR) and Russian Snow Penetrometer (RSP) Combined Testing. Glacies.

[4] Chen T., Ji L., Xiao E., Jiang C., Huang Q., Gu X.L., Qiao G. (2024). An investigation of stress analysis via finite element methods on compacted snow runways. Cold Reg. Sci. Technol..

[5] Han G., Lv Z., Zhang K., Wei H. (2026). Stereo 3D reconstruction for snow compaction and flatness monitoring in Antarctica. Measurement.

[6] Russell-Head D.S., Budd W.F., Moore P.J. (1984). Compacted snow as a pavement material for runway construction. Cold Reg. Sci. Technol..

[7] Lang R.M., Blaisdell G.L., D’Urso C., Reinemer G., Lesher M. (1997). Processing snow for high strength roads and runways. Cold Reg. Sci. Technol..

[8] Xiao E., Huo H., Dai X., Hu B., Li T., Ding J., Tang X., Sun B. (2024). Load-bearing tests and simulation analysis of compacted loose snow. Constr. Build. Mater..

[9] Schöttner J., Zeller-Plumhoff B., Hagenmuller P., Weißgraeber P., Rosendahl P.L., Löwe H., Schweizer J., van Herwijnen A. (2026). The influence of snow microstructure on the compressive mechanical properties of weak snowpack layers. Acta Mater..

[10] Huo H., Xu H., Wu J., Li T., Liu J., Xiao E., Tang X. (2026). Experimental investigation of the direct shear strength parameters of compacted snow. Cryosphere.

[11] Védrine L., Hagenmuller P., Gélébart L., Montagnat M., Löwe H. (2025). Sensitivity of the viscoplasticity of polycrystals to porosity and pore-to-crystal size ratio. Acta Mater..

[12] Wang E., Fu X., Han H., Liu X., Xiao Y., Leng Y. (2021). Study on the mechanical properties of compacted snow under uniaxial compression and analysis of influencing factors. Cold Reg. Sci. Technol..

[13] Lintzén N., Edeskär T. (2015). Uniaxial strength and deformation properties of machine-made snow. J. Cold Reg. Eng..

[14] Huo H., Jia W., Sun T., Cheng X., Chen Q., Xu H., Li T. (2025). Experimental Study on the Changing Law of Compressive Strength and the Sintering Mechanism of Compacted Snow. Mater. Rep..

[15] Wang X., Baker I. (2013). Observation of the microstructural evolution of snow under uniaxial compression using X-ray computed microtomography. J. Geophys. Res. Atmos..

[16] Hagenmuller P., Chambon G., Naaim M. (2015). Microstructure-based modeling of snow mechanics: A discrete element approach. Cryosphere.

[17] Ma C., Zheng H., Yang N., Sun T., Si J., Ren D. (2025). Microstructural evolution and mechanical properties of snow under compression. Constr. Build. Mater..

[18] Li T., Huo H., Hu B., Jia W., Chen Q. (2024). Discrete element method analyses of the polar high-density sintered snow under the unconfined compression tests. Chin. J. Polar Res..

[19] Huo H., Xu H., Xiao E., Li T., Dai X., Li R., Zhang B., Hu B. (2025). Mesomechanical analyses on the failure mechanism of the snow cluster assembly under direct shear test using discrete element method. J. Terramech..

[20] Herny C., Hagenmuller P., Chambon G., Peinke I., Roulle J. (2024). Microstructure-based modelling of snow mechanics: Experimental evaluation of the cone penetration test. Cryosphere.

[21] Schöttner J., Walet M., Rosendahl P., Weissgraeber P., Adam V., Walter B., Rheinschmidt F., Löwe H., Schweizer J., van Herwijnen A. (2025). On the compressive strength of weak snow layers of depth hoar. J. Glaciol..

[22] Xiao E., Han T., Zhang Q., Yin Z., Wang H., Hu B., Tang X., Sun B., Zhang F., Wang Y. (2026). Pressure sintering effect on the uniaxial compressive strength of reconstituted and compacted Antarctic snow. J. Glaciol..

[23] Wei J., Lu P., Han H., Wang Q., Li X., Yu M., Huo P., Li Z. (2026). Experimental study on the unconfined apparent shear strength of compacted snow. J. Glaciol..

[24] Védrine L., Brun M., Bozon M., Laurent B., Hagenmuller P. (2026). Temperature sensitivity of snow viscoplasticity: Evidence from controlled creep experiments. Cryosphere.

[25] Védrine L., Hagenmuller P. (2026). Revisiting snow settlement with microstructural knowledge. Cryosphere.

[26] Moeineddin A., Platen J., Kaliske M. (2024). Constitutive description of snow at finite strains by the modified cam-clay model and an implicit gradient damage formulation. Int. J. Numer. Methods Eng..

[27] Xiao E., Li S., Wang H., Hu B., Tang X., Sun B., Zhang F., Wang Y. (2026). Experiment on ductile to brittle transition behavior of compacted Antarctic snow under uniaxial compression. Cold Reg. Sci. Technol..

[28] Védrine L., Hagenmuller P., Gélébart L., Montagnat M., Bernard A. (2024). Role of Ice Mechanics on Snow Viscoplasticity. Geophys. Res. Lett..

[29] Miot M., Wautier A., Polfer P., Philippe P., Fülöp T., Nicot F. (2025). Mechanics of densely packed snow across scales: A constitutive modelling strategy based on the 3-D H-model. J. Glaciol..

[30] Sundu K., Ottersberg R., Jaggi M., Löwe H. (2024). A grain-size driven transition in the deformation mechanism in slow snow compression. Acta Mater..

[31] Zheng Y., Chen T., Jiang C., Huang Q., Gu X.L. (2026). Stress-rate-controlled compression behavior of compacted snow. Cold Reg. Sci. Technol..

[32] Hodgdon T., West B., Parno J., Bernier A., Courville Z. (2025). The importance of snow microstructure: An analysis of snow compressive strength under high strain. Cold Reg. Sci. Technol..

[33] Zheng Y., Chen T., Jiang C., Huang Q., Gu X.L. (2025). An explanatory model for failure modes of compacted snow under uniaxial compression. Cold Reg. Sci. Technol..

[34] Vallero G., Barbero M., Barpi F., Borri-Brunetto M., De Biagi V. (2025). An elasto-visco-plastic constitutive model for snow: Theory and finite element implementation. Comput. Methods Appl. Mech. Eng..

[35] Huo H., Hu B., Li T., Yang Y., Jia W. (2023). DEM analyses of mesoscopic failure mechanism and stress bearing mechanism of compacted snow subjected to unconfined compressive loading. Cold Reg. Sci. Technol..

[36] Wang H., Xiao E., Wang Y., Li S., Tang X., Sun B. (2025). Compaction mechanics of unsintered snow layers based on particle image velocimetry techniques. Cold Reg. Sci. Technol..

[37] Inoue R., Fujita S., Kawamura K., Oyabu I., Nakazawa F., Motoyama H., Aoki T. (2024). Spatial distribution of vertical density and microstructure profiles in near-surface firn around Dome Fuji, Antarctica. Cryosphere.

[38] Farzaneh M., Fofana I., Hemmatjou H. (2007). Effects of temperature and impurities on the DC conductivity of snow. IEEE Trans. Dielectr. Electr. Insul..

[39] Glen J.W., Paren J.G. (1975). The electrical properties of snow and ice. J. Glaciol..

[40] He J., Zheng H. (2026). Dielectric Response Characteristics and a Preliminary Ice-Type Discrimination Framework for Ice Accretion on High-Voltage Transmission Lines. Energies.

[41] Dong L., Zhang Z., Zheng H., Yang G., Hu J., Shu L., Jiang X. (2025). Ice thickness/density measurement method based on its capacitance effect. High Volt..

[42] Lemaitre J. (1985). A continuous damage mechanics model for ductile fracture. J. Eng. Mater. Technol..

[43] Weibull W. (1951). A statistical distribution function of wide applicability. J. Appl. Mech..

[44] Reiweger I., Schweizer J., Dual J., Herrmann H.J. (2009). Modelling snow failure with a fibre bundle model. J. Glaciol..

[45] Capelli A., Reiweger I., Schweizer J. (2020). Studying Snow Failure With Fiber Bundle Models. Front. Phys..

[46] Xiao E., Li S., Wang H., Hu B., Tang X., Sun B., Zhang F., Wang Y. (2026). A fiber bundle model for compressive failures of compacted Antarctic snow. Front. Phys..

[47] Moeineddin A., Wiese K., Hamad F., Platen J., Boston C., Bederna C., Kaliske M. (2026). A rate-type damage–healing Cam-Clay model for hard-packed snow: Material tests and large-scale cutting simulations by the gradient enhanced Material Point Method. Int. J. Plast..

[48] Moeineddin A., Platen J., Zhao Y., Choo J., Kaliske M. (2026). A damage–healing finite-strain Cam-Clay model for hard-packed snow. Comput. Methods Appl. Mech. Eng..

[49] Chen T., Zheng Y., Jiang C., Huang Q., Gu X.-L. (2026). Mechanical properties of compacted snow under uniaxial compression. Constr. Build. Mater..

[50] Fourteau K., Freitag J., Malinen M., Löwe H. (2024). Microstructure-based simulations of the viscous densification of snow and firn. Cryosphere.

[51] Limbrock J.K., Weigand M., Kemna A. (2025). Temperature Dependence of the Low-Frequency Electrical Properties of Partially Frozen Rocks. J. Geophys. Res. Solid Earth.

